# Diversity and functional analysis of salivary microflora of Indian Antarctic expeditionaries

**DOI:** 10.1080/20002297.2019.1581513

**Published:** 2019-02-27

**Authors:** Brij Bhushan, A. P. Yadav, S. B. Singh, L. Ganju

**Affiliations:** Defence Research and Development Organization (DRDO), Defence Institute of Physiology and Allied Sciences (DIPAS), New Delhi, India

**Keywords:** Antarctica, stress, oral microbiota, saliva, metagenome, metabolism

## Abstract

**Introduction**: The human oral microbiota continues to change phenotype by many factors (environment, diet, genetics, stress, etc.), throughout life with a major impact on human physiology, psychology, metabolism and immune system. Amongst one such factor with unique and extreme environmental conditions is Antarctica. The sea voyage to Antarctica has many risks than at station for expedition members. In this study, we investigated the influence of Antarctic sea voyage and stay at the Indian Antarctic station Maitri, on the health of Indian expedition members by using a metagenomic approach to explore oral biodiversity.

**Methods**: Saliva samples were collected from 12 expedition members, at 3 different time points viz. before the start of the ship voyage, after the completion of the voyage and at the end of the stay at Antarctica. Samples were analyzed for whole genome and 16S rRNA sequencing.

**Result**: The oral microbial diversity of the expedition members was significantly changed, during the days of sailing and after the stay at Antarctica. The oral microbiota comprised mainly of the phyla Firmicutes (46%, 29% & 36%); Proteobacteria (40%, 48%, & 44%), Bacteroidetes (10%, 22%, &14%), Fusobacterium and Actinobacteria (5%-1%) and Unclassified (17%, 25% & 23%), at three time points, respectively. Further, the differential analysis of microbes across all the phyla revealed 89, 157 and 157 OTUs genera. The altered microbiota indicated changes in amino acid, lipid and carbohydrate metabolism.

**Conclusion**: Study suggests that understanding the compositional and functional differences in the oral microbiota of Antarctic expedition members, can lay the foundation to relate these differences to their health status. It will further demonstrate the need for providing improved management during ship voyage and stay in Antarctica.

## Introduction

Antarctica, the fifth largest of the Earth’s seven continents is the southernmost, coldest, windiest, remotest and most recently discovered continent having no long history. Other than the Arctic it is hard to imagine a more harsh and unforgiving place. Seafaring to the most hostile and least populated continent on earth implies numerous environmental challenges for humans including ocean and Antarctic milieu, such as high humidity (90–100%), salinity (30–35%), UV radiation, stormy waves, blizzards, extreme low temperatures, isolation, fear, confinement, altered circadian rhythm and inadequate supply of fresh fruits and vegetables. This makes life onboard and in Antarctica unique, causing significant physiological and psychological problems. For expedition members who work under such challenging situations during the voyage and stay in Antarctica, conditions manifest into problems including seasickness, fear, gastritis, loss of appetite and sleep, etc., as compared to land-based workers [1–3]. Conditions thus required there are not only the special ships and aircrafts but also psychological balance of thoughts, which would not be accidental but deliberate. No one goes there casually. Over 100 years people have been visiting there exclusively on the basis of exploration. ‘It is a continent of extremes and of contrasts where there is no middle way’ [4]. It is also called a ‘natural laboratory’ [5]. With the advances in the technologies, much attention has been paid to the health management of expedition members during the voyage and stay in Antarctica, but yet many areas of research remain unexplored like how the extreme environment, stress, fearful journey and diet alter the gut and oral microbiota of individuals, remains unclear.

Trillions of microbes that exist in the human body are collectively known as the human microbiome which has approximately 100-fold more members than our own genome, and is thus sometimes is known as the ‘second human genome’ [6,7]. The US NIH Human Microbiome Project (HMP) (http://www.hmpdacc.org) has characterized the microbiome in five major body sites, some of which promote health and others contribute to illness [8]. It is important to understand the variations within the microbiota, to explore the impact derived from age, genetics, stress, fatigue, routine behaviour, dietary changes, sanitary and living conditions, smoking, antibiotics and most of all the environment [9–13], on these complex, commensal and poorly understood communities. The findings can be extended to understand the interactions between the host and the microbiota and its impact on human health.

It is known that mucosal surfaces are the main barriers to the environment [14]. The most important, the oral mucosa, is considered to be one of the effective hindrances to the outside world and also an immunological and biochemical organ [15]. As described by Pennisi the human mouth is a portal of entry for pathogen to the respiratory and gastrointestinal tracts, and it is very important to understand the constitution and dynamics of microbial communities reflecting the entire health status [16]. The Human Oral Microbiome Database (HOMD) reveals that oral mucosal immunity in coordination with oral commensal organisms maintain the homeostasis of the oral cavity (http://www.homd.org), and play a fundamental role such as metabolism and physiological functions in the well-being of the host due to severe environmental factors and diet changes [17]. Physical activities and the interruption of the normal circadian rhythm can also influence the oral microbial diversity due to the fluctuating pattern of the biological clock. Jet lag-induced dysplasia and diurnal fluctuations could be causally related to metabolic abnormalities, such as glucose intolerance and obesity [18]. Abnormality of the oral microbiota has been reported to be correlated with many diseases, such as periodontitis, pneumonia, acute postinfectious glomerulonephritis, septicemia, rheumatic fever, pre-term birth, cardiac health as well as cognitive function, because of some pathogens residing in the throat and saliva [19,20]. In the medical reports available for the past seven Indian scientific expeditions to Antarctica, dental conditions were the commonest diseases (25%), followed by conditions of the digestive tract such as diarrhea and dyspepsia [21]. The finding of major periodontopathic bacteria in non-dental sites, especially on the tongue, may argue for antimicrobial treatment not only for the dental biofilm but for the entire oral cavity [22]. The human microbiome has come to the rescue of many conditions, which remained untreated with conventional methods. Monitoring of the oral microbiota could be a useful tool for early diagnosis of many diseases.

In this study, we investigated the oral commensal microbes and the functional association between the oral microbiota and the host health status using a metagenomics approach, for Antarctica expeditionaries, who performed a voyage in the Indian ocean for 25 days and thereafter stayed in Antarctica for more than 30 days. DNA-based whole genome sequencing and 16S rRNA phylotyping were combined for identification of major bacterial phyla for altered microbial diversity and functional analysis for annotation pertaining to microbial nutrient metabolism for various pathways.

## Materials and methods

### Ethics statement and volunteer information

All expedition participants understood the nature of the study and gave their written consent. The Ethics Committee of the Defence Institute of Physiology and Allied Sciences, the Defence Research and Development Organisation, New Delhi, India, approved all the relevant parameters of the study. The study protocols were in accordance with the approved guidelines.

### Study design

A total of 25 volunteers participated in the 34^th^ Indian Scientific Summer Expedition to Antarctica (ISEA), out of which 12 volunteered to the present study. The demographic data of all participants were collected at the beginning of the study (Table 1). The expedition team stayed in the Indian Antarctic Station, Maitri (70▫45′E, and 11▫44′S), located in the Central Dronning Maudland region of east Antarctica, about 100 km inland from the Princess Astrid coast. The expedition team was selected on the basis of interviews and some criteria set by authorities suitable for the expedition. The participants were subjected to thorough medical and psychological examinations to ensure a healthy population, followed by an acclimatization and orientation course at the high-altitude location Auli, Uttarakhand, in the Indian Himalayas. Participants took the voyage to Antarctica for 25 days one way, covering a distance of about 6,816 nautical miles. All the participants were males aged 22–55 years; neither had any signs or symptoms indicative of infection during the study nor did they use any drugs that could significantly affect the general health.

**Table 1.** Demographic data for the 12 patients participating in the study.

Parameters	Values (*n* = 12)
Age, yrs median (IQR)	32.5 (28.0–37.0)
Weight, kg, median (IQR)	75 (72.5–82)
SBP, mmHg, median (IQR)	120 (110.5–128.5)
DBP, mmHg, median (IQR)	74 (59.7–80.0)
Pulse rate, per min., median (IQR)	64.5 (59.0–75.5)
SpO2, %, median (IQR)	99 (98–99)
Respiratory rate, per min, median (IQR)	17 (14.0–18.75)
Mean Arterial pressure, mmHg, median (IQR)	95.17 (89.33–108.2)
Food Habit, %	
Veg: non-veg	16.66: 83.3
Smoking, %	33.33
Alcohol, %	83.33
Sea Sickness, %	
T1	–
T2	66.6
T3	75

Abbreviations: **yrs –** Years; **IQR –** Interquartile range; **Kg –** Kilograms; **SBP –** Systolic blood pressure; **mmHg –** Millimeter of mercury (Hg)**; DBP –** Diastolic blood pressure; **SpO2 –** Peripheral capillary oxygen saturation; **Veg –** Vegetarian; **non-veg –** Non-vegetarian; **T1 –** Time point 1**; T2 –** Time point 2**; T3 –** Time point 3.

#### Sample collection

Passive saliva samples (approximately 2 ml) were collected between 7 and 9 am, without consumption of any food or water, prior to sample collection at three different time points of the entire expedition. The first one was for baseline data collection, at sea level off Cape Town (T1) two days prior to the beginning of the voyage to Antarctica; the second was on board after completion of 25 days of voyage (T2) without any sea shore stoppage, and the third and final collection was at the end of 30 days of stay (T3) at Maitri. Immediately after the collection, samples were frozen at – 22°C without culturing and finally transferred to a –80°C freezer before processing.

### Sample preparation and DNA extraction

Two ml saliva collected in a Falcon tube was diluted with 4 ml PBS and centrifuged at 1,800 x g for 5 min. Genomic DNA was isolated from the pellet using the QIAamp DNA Mini Kit (Qiagen, Hilden, Germany). The quantity and quality of isolated DNA were measured using a Nano Drop ND-1000 spectrophotometer (Thermo Fisher Scientific, Waltham, MA) and agarose gel electrophoresis, respectively. All the extracted DNA samples were normalized and then stored at −20°C until further use.

### 16S v3-v4 amplification, library preparation and sequencing

16S rRNA sequencing was conducted on an Illumina MiSeq platform. To amplify and sequence the V3-V4 hypervariable regions of the 16S rRNA gene, the 341F and 805R (Table 2) universal primers were used targeting a region of approximately 464 bp encompassing variability [23].

This region provides sample information for classification and identification of microbial communities from specimens associated with human microbiome studies in line with the Human Microbiome Project. The reverse primer contains a 6-bp error-correcting barcode unique to each sample. The final amplified amplicon libraries were purified using AMPure XT beads (Beckman Coulter Genomics, MA) and the size and quantity of the amplicon library were assessed on the Bioanalyser (Agilent technologies, USA) and the Library Quantification Kit for Illumina (Kapa Biosciences, MA), respectively. The PhiX Control library (V3) (Illumina) was combined with the amplicon library (expected at 20%). The library was clustered to a density of approximately 570 K/mm^2^, and the libraries were sequenced through 250PE (Paired end) Illumina MiSeq runs. Sequencing data were available within approximately 48 h. Image analysis, base calling and data quality assessment were performed on the MiSeq instrument.

### Genomic DNA pooling with respect to different time points and WGS shotgun library preparation and sequencing

To assess the changes at gene and pathway levels, across different time points during the long voyage, the genomic DNA samples at a particular time point were pooled in equimolar concentrations. From this pooled and purified metagenomic DNA 0.5 μg was shared and a paired-end library with an insert size of ~300 bp was constructed for each sample. A shotgun library was then constructed according to a standard True Seq WGS (whole genome sequencing) protocol provided by Illumina, Inc. (San Diego, CA). Quantification was performed using a Qubit Fluorometer (Invitrogen, Life Technologies, Grand Island, NY) and a Stratagene Mx3000P Real-time PCR Cycler (Agilent, Santa Clara, CA) prior to cluster generation in a c-Bot automated sequencing system (Illumina, Inc.). Three libraries with different indices were pooled together and sequenced in one lane using an Illumina HiSEQ 2500 high-throughput sequencing instrument with 2 × 75 bp paired-end (PE) sequencing.

### Quality check (QC), operational taxonomy unit clustering and taxonomy assignment

The 16S analysis was performed using CLC microbial Genomics Module v2.0 (Qiagen, Valencia, CA). Quality filtering consisted of discarding reads <200 bp and >1,000 bp, excluding homopolymer runs >6 bp and ambiguous bases >6 bp, accepting one barcode correction and two primer mismatches. A value of 25 mer was considered as the minimum average. Phred quality score allowed in reads in a sliding window of 50 bp followed by hos sequences removed (Homosapiens hg 19). Paired reads were merged using the Optional Merge Paired Reads tool at default parameters-mismatch: 2, mismatch score: Default 8 (an overlap of 11 bases with one mismatch), gap cost: 2 (an insertion or deletion in the alignment). For clustering the merged sequences, all reads were trimmed to the same length using the fixed length-trimming algorithm and calculated as the mean length of the merged reads minus one standard deviation for the combined reads in all samples [24]. Finally, the operational taxonomic units (OTUs) clustering tool clustered fixed length trimmed reads to OTUs at 97% similarity. Chimeras were removed, generating abundance of the OTUs for all samples (default abundance 10), with Singleton OTUs removed for statistical analysis. The analysis tool used for taxonomy assignment was performed employing the naïve Bayesian RDP classifier with a minimum confidence of 0.8 against the Green genes database resulting in the names of the identified taxa (assemblies).

### 16S rRNA functional prediction and WGS functional metagenome

For 16S rRNA datasets, the Phylogenetic Investigation of Communities by Reconstruction of Unobserved States (PICRUSt) software package was used for predictive functional analysis. Template-guided multiple sequence alignment (MSA) was performed using PICRUSt (v. 1.0.0) against the multiple alignment of the Greengenes [25] database (release 13_05) filtered at 97% similarity for bacterial sequences.

For WGS Metagenome data from pooled Intra-time point samples, the data were first quality filtered using FASTQC v0.11, followed by analysis pipeline in CLC Microbial Genomics Module v2.0 and Metagene mark Plugin for Annotation. De-novo assembly was performed at k-mer (k = 21, 41 and 61) in longer contigs mode with a minimum contigs length of 200 bp. Metagenemark Ab-inito gene prediction algorithm was used for annotating coding sequence (CDS) regions in the resulting contigs. The identified CDS were annotated using three databases; the NR-Protein database, the P-fam domains and the GO database [26–28] with BLAST Cutoff (10E-5). Using the functional annotation of these collected BLAST hits, we applied a majority-rule consensus approach to determine the function of the query proteins. For taxonomic annotation, we computed the lowest common ancestor (LCA) of all species in the collected BLAST hits to determine its taxonomic origin.

### Statistical analysis 16S rRNA and WGS datasets: alpha and beta diversity (PcoA), differential abundance and PERMANOVA

The merge abundance table tool was used to merge all the individual abundance tables of different samples for differential abundance and Beta diversity (PcoA) analysis. Differential abundance was estimated using the Generalised Linear Model (GLM) with Negative Binomial distribution. Significant abundances between group pairs were identified using the Wald test (Fold change >2 and p value – 0.05), and the likelihood ratio test across groups (ANOVA-like) comparison for multi-sample corrected p-values (Bonferroni corrected).

OTUs were aligned using Multiple sequence comparison by Log-expectation (MUSCLE), to create alignment used to reconstruct a Maximum Likelihood Phylogeny tree against the Greengenes database (core set aligned sequences v.2010), and the tree was generated using FastTree [29]. Rarefaction was calculated by sub-sampling the abundances in the different samples at different depths. Alpha diversity was estimated with measures calculating richness and diversity. Richness indices, Chao1 estimator [30] and abundance-based coverage estimator (ACE) [31], were calculated to estimate the number of observed OTUs present in the samples. The diversity within each individual sample was estimated using the nonparametric Shannon diversity index [32], and Simpson’s diversity index (Figure 2(e)). Multivariate analysis of community structure and diversity was performed on the datasets using: 1) Principal Coordinate Analysis (PCoA) [33], and 2) a permutation test for assessing the significance of the constraints and permutational multivariate analysis of variance (PERMANOVA). The differences between bacterial communities were investigated using the Bray–Curtis dissimilarity distance (D-05 Unifrac for robust trade-offs between rare and abundant Lineages) [34]. The null hypothesis of no differences between defined groups (*i.e*. assuming no constraints, as for the PCoA) was investigated using the PERMANOVA approach (p value-0.05) [35], and applied to the Bray–Curtis dissimilarity distance (No. of permutation: 99,999).

### Correlation between 16S rRNA PICRUSt prediction and WGS metagenome

Functional categories and their relative abundances at three different time points were compared using Wilcoxon test for paired time points (16S rRNA grouped and WGS pooled time points). WGS reads after shotgun analysis were annotated to Kyoto Encyclopedia of Genes and Genomes (KEGG) orthologies (KOs) using v0.98 of HUMAnN31. PICRUSt generated functional KOs predictions, using the 16S-based OTU tables, were compared to the annotated WGS metagenome across all KOs using Spearman rank correlation. Statistical tests used in the study were two-sided, and a p value of 0.05 or less was reported as statistically significant.

### Online data availability

All the raw data of WGS and 16S rRNA sequencing have been submitted to the NCBI sequence read archive (SRA) repository for the research community, bio-project ID PRJNA419274.

## Results

The oral microbiota of 12 members (36 saliva samples) of 34^th^ ISEA, collected at T1, T2 and T3 time points, were analyzed by performing 16S rRNA and WGS to illustrate the microbial diversity during 25 days of voyage and 30 days stay in Antarctica.

### Sequence data of the oral commensal microbiota metagenome

**Figure 1.**
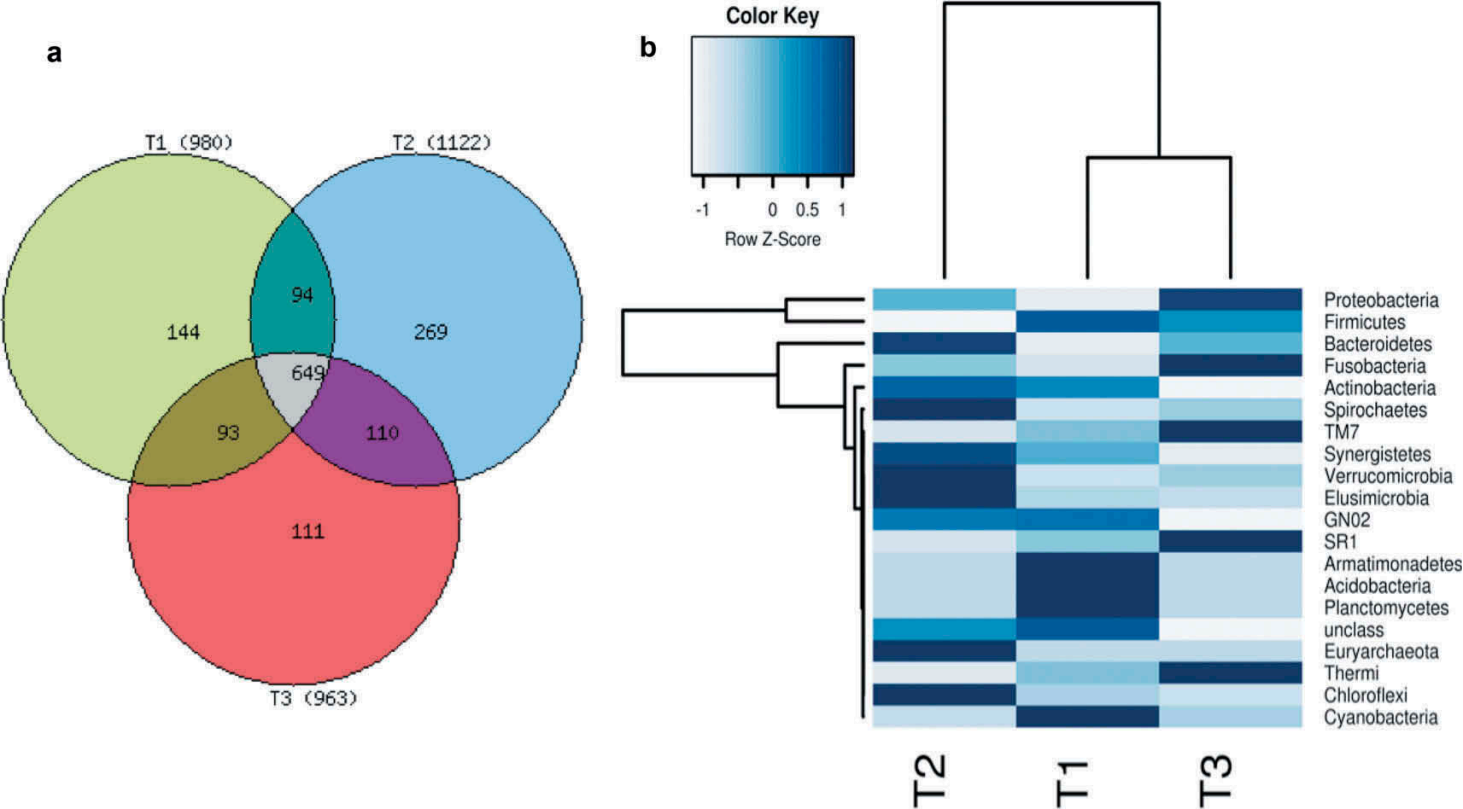
(a) represents the number of texas, Neon Green represents (T1), blue (T2), red (T3), share texas green (T1-T2), purple (T2-T3) brown (T1-T3) and gray (T1-T2-T3). (b) shows the fold changes in phyla at T1, T2 and T3 time points, dark blue represent maximum ‘Z’ score and white minimum.

We sequenced the V3-V4 regions of 16S rRNA from 36 saliva samples. All of the sequences were classified into different OTUs at 97% similarity. In total, 4,564,450 sequence reads were obtained from the 36 samples, with an average read length of 153 bp (Supplementary Data). The read number per sample ranged from 75,964 to 170,860 averaging 126,790. The rarefaction curves indicated that the sequencing coverage was adequate (Supplementary Table 1). Total taxa present in the 36 samples were 3,065 and at least two-thirds of the taxa were considered common. Among the 980, 1,122 and 963 representative OTUs found at T1, T2 and T3, respectively, 94 and 93 were shared taxa between T1 and T2, and T1 and T3, respectively (Figure 1(a)), whereas 110 were shared between T2 and T3. To obtain the phylogenetic classifications of the metagenomic reads for each sample, we performed a classification analysis using the Greengene 13.5 database and fold changes in phyla at the T1, T2 and T3 time points (Figure 1(b)). The results were assigned at phylum and genus levels based on an identity level of 97% similarity. At all three time points, a total of 47 phyla and 400 genera were obtained and annotated.

### Oral diversity analysis

**Figure 2.**
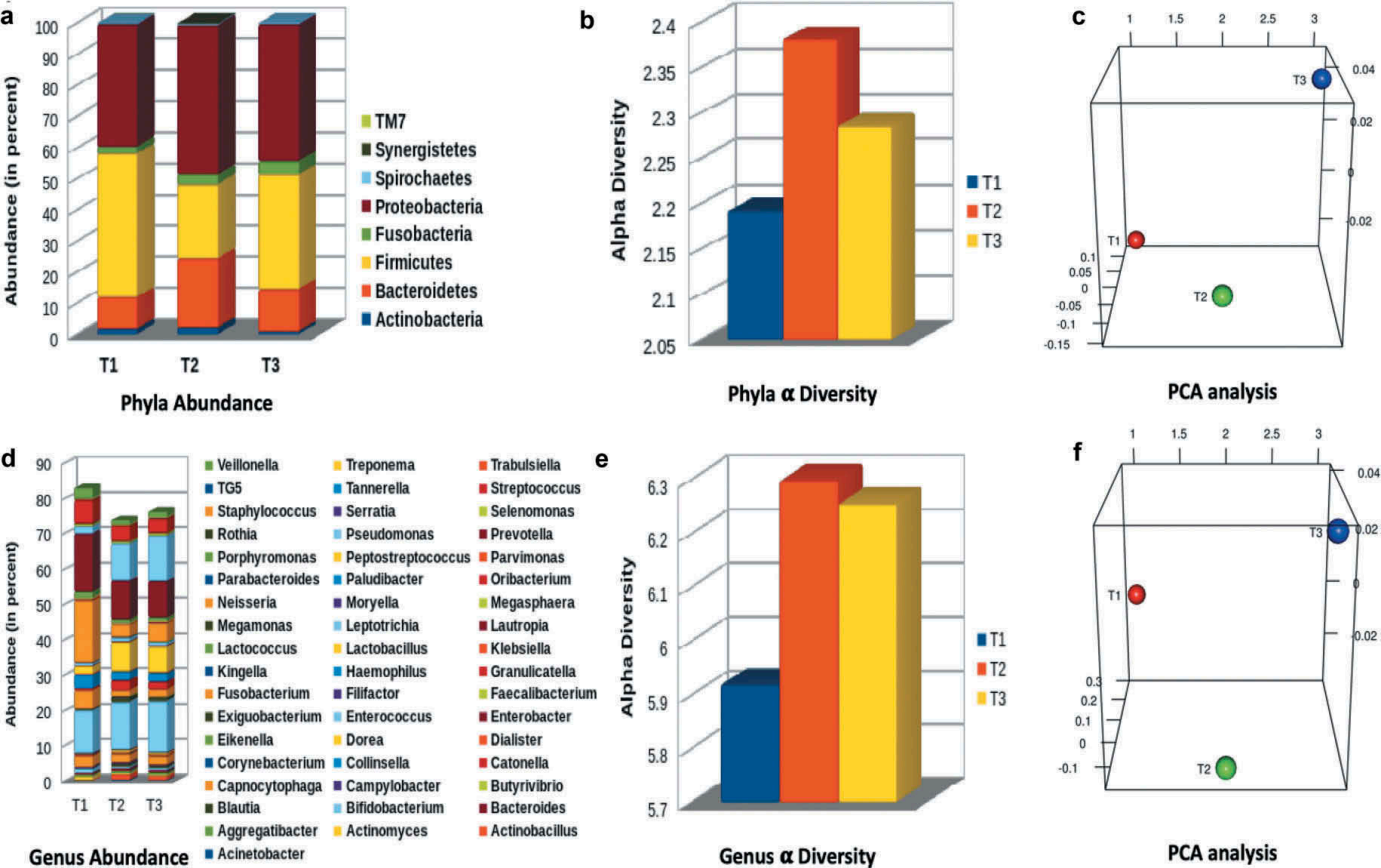
(a and d) show oral microbial abundance at phyla and genus level at three time points; (b and e) depict alpha diversities as antilog of Shannon index and (c and f) indicate beta diversity. All the data have *p* < 0.05.

The oral microbiota of 12 individuals compared at T1, T2 and T3 time points, revealed clear distinction in the microbiota through PCoA (Figure 2(c)). Three indices (Chao1, Simpson and Shannon index) employed to estimate the alpha diversity at different time points, demonstrated an increase in diversity during the 25 days of voyage and after 30 days of stay in Antarctica (Figure 2(b)).

### The taxonomic analysis at phylus level

The structure of the oral microbial community was analyzed by measuring OTUs by 16S rRNA sequencing, with known sequences at phylus level more than 1%. Firmicutes was the most predominant phylum, accounting for 46% of the identified taxa at T1. The second richest phylum was Proteobacteria with 40% presence. The next detectable phyla were Bacteriodetes 10% and Fusobacteria and Actinobacteria, 2% each, whereas, Spirochaetes, Cyanobacteria, TM7, Synergistetes and GNO2 accounted for less than 1% (Figure 2(a)). After 25 days of voyage to Antarctica *i.e*. at T2, there was a significant increase of Proteobacteria, to 48%, 22% Bacteriodetes and 3% Fusobacteria while Actinobacteria remained the same, 2% (Figure 2(a)). All other phyla were reduced after the voyage including the Firmicutes 28.80%. An interesting phenomenon was observed after staying in Antarctica for 30 days *i.e*. at T3, the predominance of microbiota was reversed and increased to 36% Firmicutes, 5% Fusobacteria and decreased to 44% Proteobacteria, 14% Bacteroidetes and 1% Actinobacteria, and others to less than 1%. The phyla abundance clearly indicated a significant change in the microbiota (Figure 2(a)).

### The taxonomic analysis at genus level

Next, we analyzed the frequency of the most abundant genera. At the genus level *Neisseria* was the most abundant genus (17.64%) at T1, followed by *Enterococcus* (12.19%), *Streptococcus* (6.64%), *Fusobacterium* (5.2%), *Haemophilus* (3.9%), *Capnocytophaga* (3.12%) *Veillonella* (3.1%), *Lactobacillus* (2.26%), *Pseudomonas* (2.17%), and others less than 1%, whereas unclassified genera were 16.82% (Figure 2(d)). Interestingly, after completion of the voyage at T2, the richest genus was *Enterococcus* with a marginal increase to 13.53%, followed by *Pseudomonas* and *Lactobacillus*, which increased significantly to 10.49% and 8.33%, respectively, compared to T1. On the contrary, genera *Neisseria* (3.51%), *Streptococcus* (4.24%), *Granucatella* (2.86%), *Haemophilus* (2.23%), *Capnocytophaga* (2.27%), *Fusobacterium* (1.70%) and *Veillonella* (1.28%), were significantly reduced. Interestingly, the unclassed genera increased to 25.43% (Figure 2(d)). Following the same trend at T3 the genera showing further marginal enhancement were *Enterococcus* (14.57%), followed by *Pseudomonas* (12.83%), *Neisseria* (5.24%) *Fusobacterium* (2.13%), *Haemophilus* (2.34%) and *Veillonella* (1.61%) whereas, genera *Streptococcus* (4.11%) and *Lactobacillus* (7.38%) decreased marginally. The unclassified genera fell to (23.17%). Other genera represented less than 1% (Figure 2(d)), while the different genera after the voyage and stay in Antarctica, mostly belonged to *Enterococcus, Pseudomonas, Lactobacillus* and *Streptococcus*.

### Functional characterization, correlation between 16S rRNA predicted and WGS sequenced functional profile

#### Predicted functional potential at different time points

**Figure 3.**
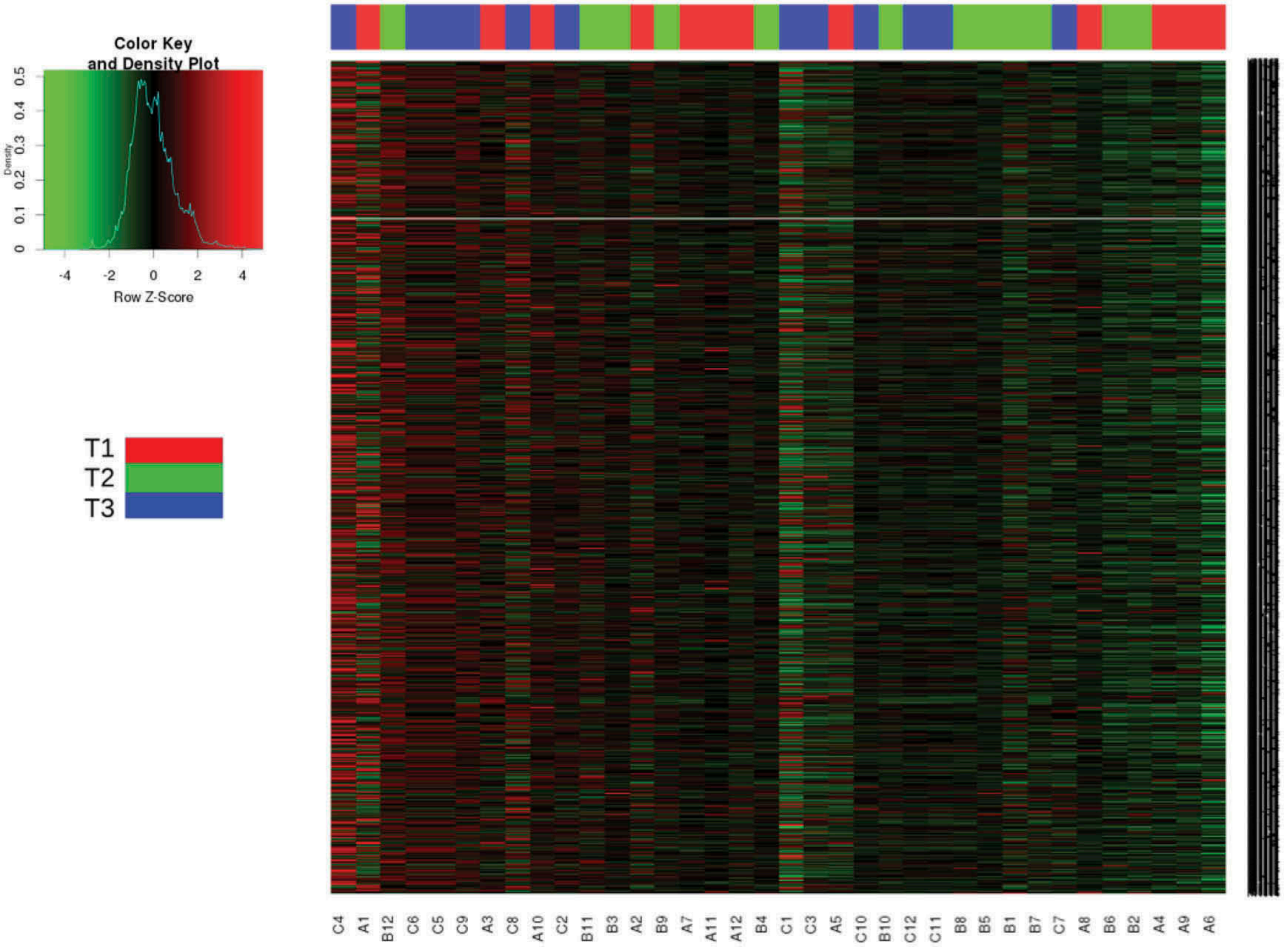
KEGG orthologs (KOs) in saliva microbiota in all individuals at different time points. The heat map shows the relative abundance of significant (*p* value <0.01) individual KEGG orthologs (Kos) calculated for time points T1, T2 and T3 samples using PICRUSt. Samples were clustered using the Euclidean distance measure.

**Figure 4.**
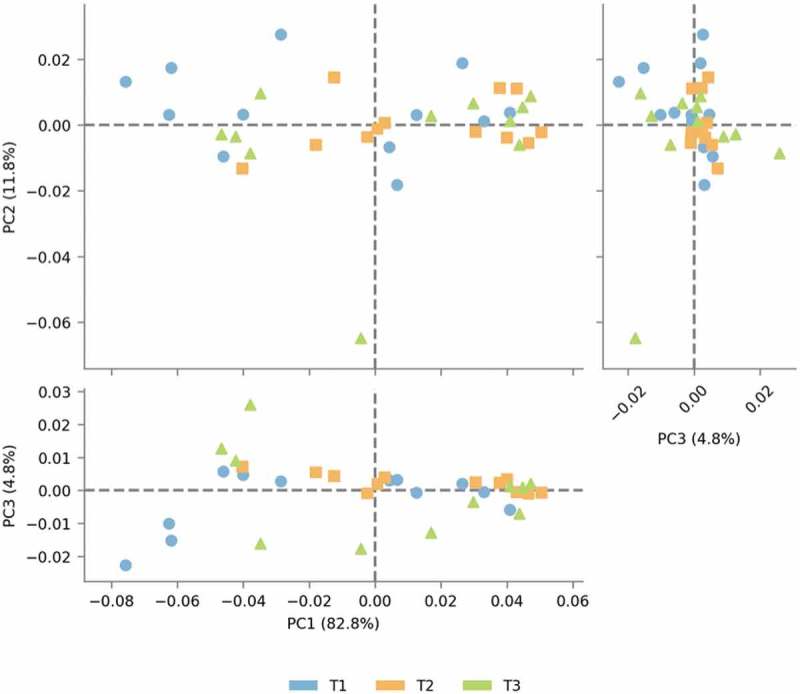
Principal coordinate analysis (PCoA) using STAMP v2.1.3 of predicted functional metagenomes between Time points T1, T2 and T3.

In addition to the community structure analysis during the long voyage, we analyzed the functional component between the time points to determine the relationship between the microbiota structure and functions involved in maintaining homeostasis and adaptation during the course of the voyage and changing landscapes, by employing a computational method PICRUSt [36]. This was done to predict the metagenomic contribution of the observed microbial community at different time points. Similar to the OTU profiles observed in our samples, comparison of the predicted KOs demonstrated significant clustering at different time points. The relative abundance of KOs present at time point T2 and T3 was predominantly increased compared to that at T1 (Figure 3). Further, STAMP v2.1.3 generated PCoA of predicted functional metagenomes showed a sparse distribution in T1 individuals on the left of the PC2 Axis, contrary to the T2 and T3 cluster on the right side of the PC2 axis (Figure 4).

**Figure 5.**
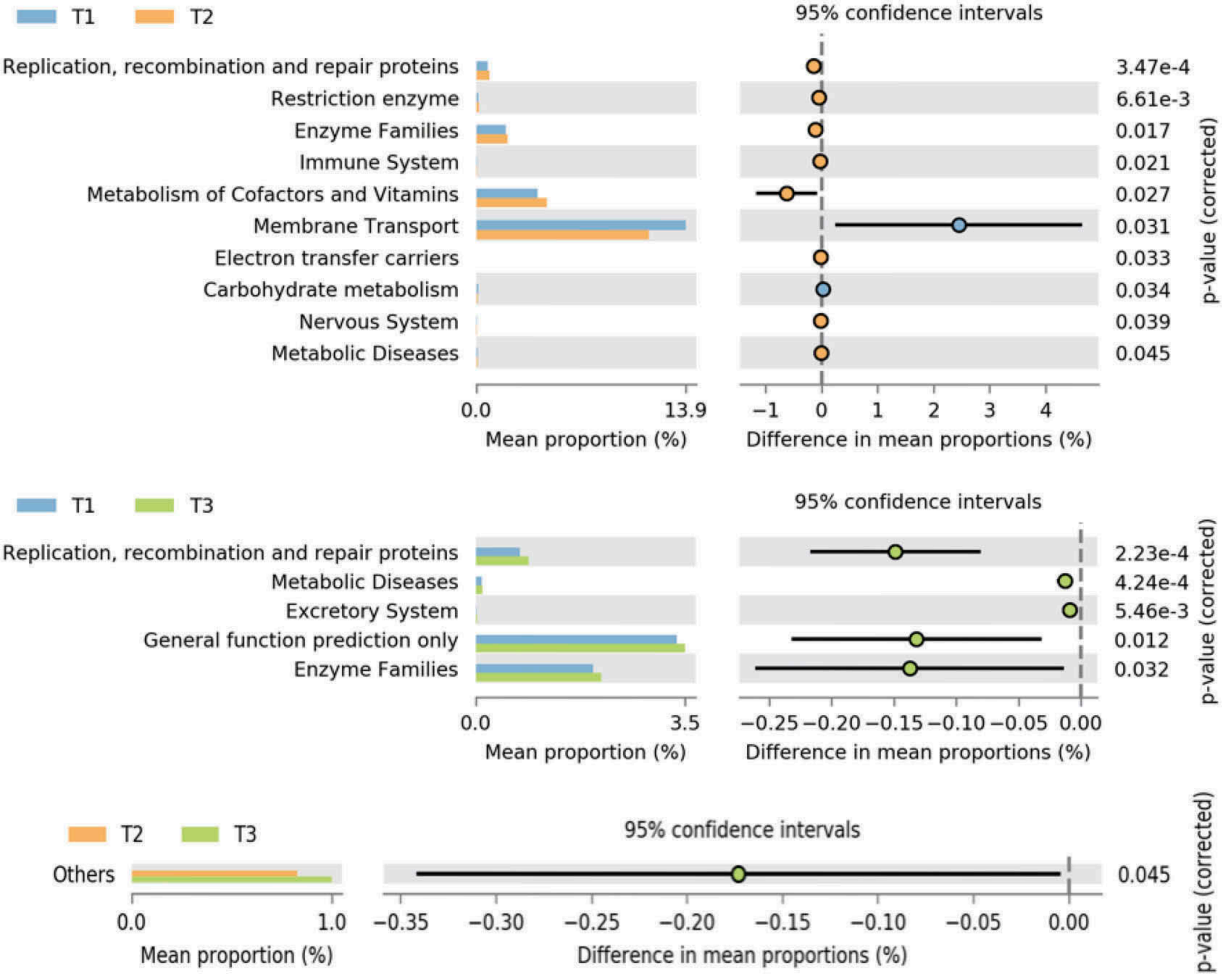
Metagenomic functional predictions pair-wise comparison between the time points (T1 – T2), (T1 – T3) and (T2 – T3) for mean relative gene pathway abundance of significantly differentially abundant modules (ANOVA; *p* < 0.01).

To encompass the major functional categories covered by individual KOs, we determined the overall gene pathways and module abundance using the HUMAnN (HMP Unified Metabolic Analysis Network). The results using statistical analysis of metagenomic profiles (STAMP) v2.1.3 were analyzed at the second tier functional categories. Among these categories, the functional abundance of Replication, Recombination and Repair proteins (p = 3.47e-4 and p = 2.23e-4), Enzyme families (p-0017 and p-0.032), Immune system (p-0.021), Metabolism of cofactor and Vitamins (p-0.027), General function Prediction (p-0.012), and Metabolic Diseases (p-0.045, 4.24e-4) were significantly increased at T2 and T3 compared to T1, whereas the membrane transport (p-0031) was significantly decreased at T2 and T3 in comparison to the T1 time point (Figure 5).

### Correlation between 16S rRNA predicted functional profile and WGS gene function profiles

**Figure 6.**
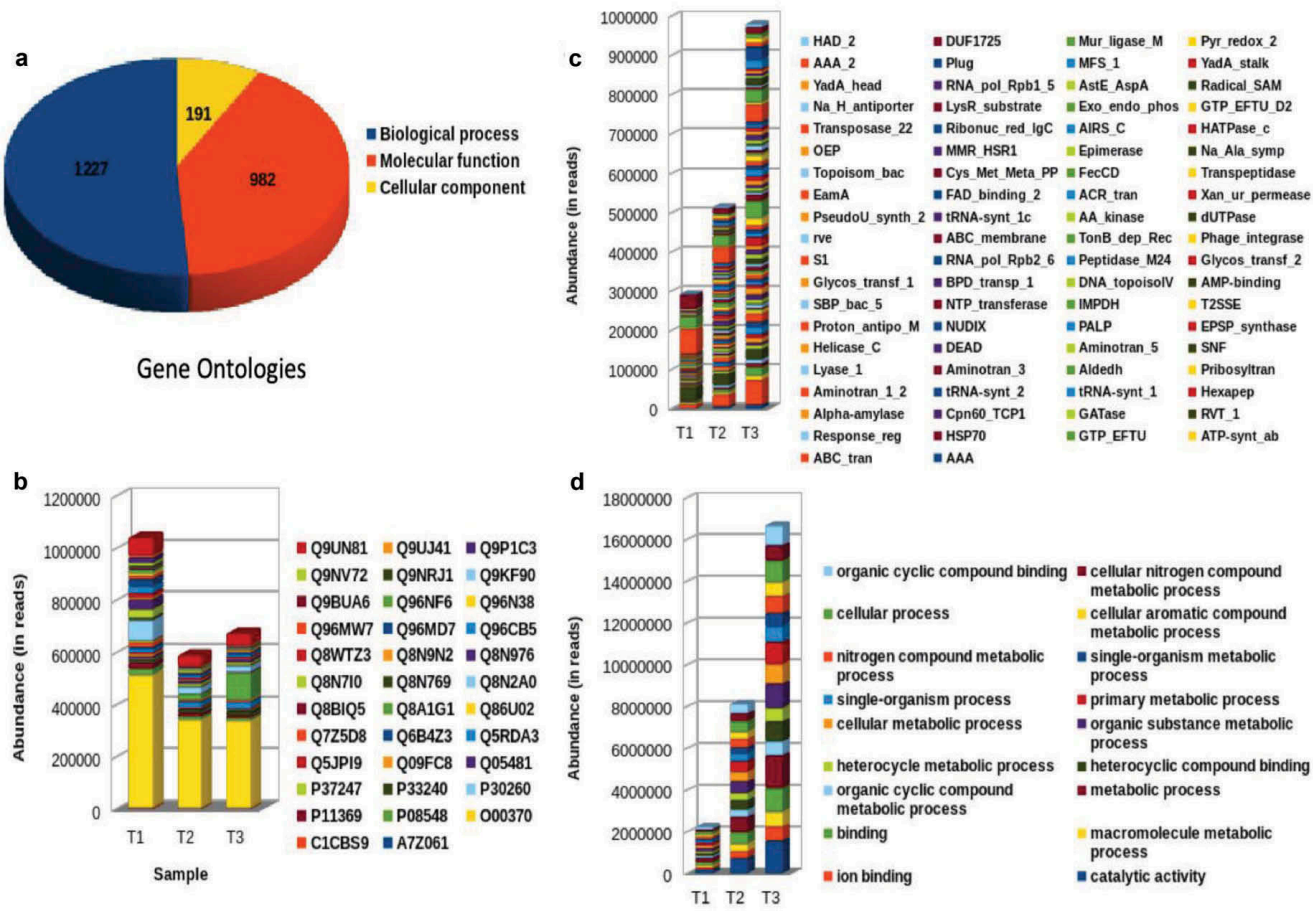
Representation of the CDS contigs annotation against (a) GO terms identified across T1, T2 & T3; (b) NR-protein; (c) GO blast and (d) Pfam abundance at various time points.

WGS raw data generated on the Illumina NextSeq 500 2 × 75 base read length were 14.3, 16.5 and 17.3 million reads at T1, T2 and T3, respectively. De novo assembly metagenome tool was used for whole metagenome analysis (beta version) of the complete linkage clustering (CLC) genomics workbench. Microbial genomics Module, generated at k-mer 21, an N50 contigs of 525 base and a total number of 304,525 sequences, covering 155 mb of the total bases. After coding DNA sequence (CDS) annotation using Metagenemark, the CDS contigs annotation was performed against the NR-Protein database (28.3%–87,843), the PFAM domains (28.98%–89,916) and the GO database (18.13%–56,264) at a BLAST Cutoff of (10E-5) (Figure 6). Correlation analyses were performed to evaluate the relationship between percent abundances of the second-tier functional gene categories in the shotgun metagenomic dataset and the percent abundances of genes inferred from PICRUSt. Site-specific differences in abundances of functional assignments were observed using PICRUSt, despite normalization to 3,52,108 sequence reads, with the log number of inferred functional assignments ranging from 8.06 to 8.76 per site. These differences in numbers of assignments are likely due to differences in copy number among the species identified at each site. No functional traits among, Cell motility, Signalling molecules and Interaction, Xenobiotics biodegradation and metabolism, Metabolism of Amino Acid and Carbohydrate and Enzyme families, were positively correlated between shotgun metagenomic and PICRUSt datasets. The percent abundances of all second-tier functional traits were not significantly different between datasets via ANOVA (p ≤ 0.3333). Among traits that differed by >5% between the methods, Amino acid metabolism and Translation were higher in the shotgun metagenomic dataset, while PICRUSt inferred greater abundance of membrane transport (Table 3).

**Table 2.** Forward and reverse primer sequence of V3-V4 region of 16S rRNA.

V3–V4	Illumina_16S_341F	5′ -TCGTCGGCAGCGTCAGATGTGTATAAGAGACAGCCTACGGGNGGCWGCAG
	Illumina_16S_805R	5′-GTCTCGTGGGCTCGGAGATGTGTATAAGAGACAGGACTACHVGGGTATCTAATCC

**Table 3.** List of 1st tier, 2nd tier and 3rd tier pathways with their metagenome and Picrust prediction along with spearman (r) correlation values and the genus with may be responsible for the alteration in pathway.

1st Tier	2nd Tier	3rd Tier	Metagenome(%)	picrust(%)	Spearman (r)	p value	Genus
Metabolism	Metabolism of Cofactors and Vitamins	Biotin metabolism	0.762	1.675	2	0.110	*Fusobacterium, Haemophilus, Neisseria, Pseudomonas*
		Riboflavin metabolism	0.380	3.885	2	0.068	*Fusobacterium, Haemophilus, Pseudomonas, Streptococcus*
		Thiamine metabolism	0.604	2.436	2	0.186	*Neisseria, Streptococcus*
		Nicotinate and nicotinamide metabolism	0.762	3.561	4	0.496	*Pseudomonas*
		Ubiquinone and other terpenoid-quinone biosynthesis	0.831	0.089	2	0.456	*Neisseria, Pseudomonas*
	Metabolism of Other Amino Acids	D-Alanine metabolism	0.005	2.769	6	0.296	*Pseudomonas, Streptococcus*
	Lipid Metabolism	Fatty acid biosynthesis	8.707	0.621	6	0.454	*Haemophilus, Neisseria, Pseudomonas, Streptococcus*
		Fatty acid elongation in mitochondria	0.292	2.219	0.535	0.187	*Pseudomonas*
		Fatty acid metabolism	0.292	2.982	7.464	0.124	*Pseudomonas*
	Carbohydrate Metabolism	Galactose metabolism	0.388	13.077	6	0.223	*Fusobacterium, Neisseria, Streptococcus*
		Glycolysis/Gluconeogenesis	0.388	3.064	6	0.032	*Fusobacterium, Haemophilus, Neisseria, Pseudomonas, Streptococcus*
		Pyruvate metabolism	3.586	2.641	6	0.480	*Streptococcus*
	Lipid Metabolism	Lipid biosynthesis proteins	0.625	5.301	2	0.062	*Haemophilus, Neisseria, Pseudomonas*
	Glycan Biosynthesis and Metabolism	Lipopolysaccharide biosynthesis	37.164	5.077	2	0.002	*Fusobacterium, Haemophilus, Pseudomonas*
		Lipopolysaccharide biosynthesis proteins	0.945	2.110	2	0.001	*Fusobacterium, Haemophilus, Pseudomonas*
		Peptidoglycan biosynthesis	8.707	2.726	4	0.001	*Pseudomonas*
	Xenobiotics Biodegradation and Metabolism	Toluene degradation	4.613	4.744	4	0.002	*Pseudomonas*
Organismal Systems	Digestive System	Protein digestion and absorption	4.316	1.360	0	0.019	*Haemophilus, Neisseria, Pseudomonas*

## Discussion

The present study describes the oral metagenomic analysis of the participants of 34^th^ ISEA, spending 25 days on a voyage and 30 days in Antarctica with extreme climatic conditions under which individuals express a series of physiological and psychological symptoms that manifest into various health problems and declined physical performance. For better survival, stability and performance, it is very important to have diversity in all microbial ecosystems [37]. The more diversity, the higher resiliency. Alteration in the external biodiversity is directly proportional to the human commensal microbiota and its correlation with other metabolic pathways and immunity of the body [38,39]. With the advent in technology, the metagenome is a tool extensively studied to understand the effect of altered microbiome on physical conditions, and its impact on human health [40].

The human oral microbiota is under direct attack by the external environment. Zheng et al. have reported reduced microbial diversity in the oral cavity and belly button during long voyages of 120 days, confirming the influence of exposure to a stressful voyage environment on the human body [41]. The Antarctic milieu has multiple stresses including torturous voyage, adverse temperature, inappropriate nutrition, sleep deprivation, etc., causing severe physiological and psychological stresses [42,43]. To our knowledge, this is the first study to report the effect of voyage and stay in Antarctica on the oral microbiota of Indian expedition members belonging to a spectrum of age groups and ethnicity by 16S rRNA and WGS. As we intended to study a sample representative of the population the exclusion criteria included only the most frequently reported conditions that affect the healthy oral microbiome such as wearing dentures, mouth sprays, pregnancy, reduction of the salivary flow, etc..

The oral microflora is the second most complex microbiota of the body, colonizing in the abundance of approximately 1,000 species (Human Microbiome Project 2012). In our study, the bacterial abundance varied with the conditions at different time points. The most abundant phyla before the start of the voyage (T1) was Firmicutes (46%) (previously referred to as the low-G + C Gram-positives, with genera like *Streptococcus, Enterococcus, Lactobacillus, Veillonella* and related ones), followed by Proteobacteria (40%) with genera *Neisseria, Haemophilus, Pseudomonas*, and related ones), which subsequently increased during the voyage (T2) and stay in Antarctica (T3). This alteration in phyla indicated the impact of a stressful environment on the oral microbiota. Consequently, the predominant genera at T2, the *Pseudomonas* and *Lactobacillus* followed by *Enterococcus*, are generally considered as transient oral bacteria [44,45], related to oral and respiratory diseases, e.g. caries, endodontic infections and periodontitis [46–48]. Lactobacilli demonstrate an antagonistic action against periodonto-pathogens such as *Prevotella intermedia, Porphyromonas gingivalis* etc., inhibiting their growth and resulting in production of lactic acid lowering the pH of the environment and release of H_2_O_2_ and bacteriocins, thus maintaining the micro-ecological balance in the oral cavity [49–51]. A very interesting phenomenon was observed when the abundance of unclassified sequences increased both during the voyage as well as the stay in Antarctica. This provides an opportunity to further explore and understand the Antarctica environment and its implications on human health and microbiota.

Expedition participants often had dry mouth, increased respiratory rate and inadequate fluid intake because of cold and dry winds. Oral hygiene often becomes a secondary consideration when people are tired and exposed to extreme cold/heat which makes teeth sensitivity a major issue. As a result of this, teeth may be subjected to trauma while consuming cold and frozen stuff, e.g., frozen chocolate, a common culprit, which is part of the expedition diet. Auburn, as noticed in his personnel, commented on oral hygiene: ‘the toothbrush remains for most rather an instrument for use on ceremonial occasions’ [52]. Risk of oral problems includes physical, biological, environmental, behavioral and lifestyle-related factors [53], which become an important issue under a stressful environment. In the 27^th^ ISEA, oral ulcers observed in the early part of the winter period, though mild in nature, were treated by using oral anesthetic gels, multivitamin capsules, and oral hygiene measures. The incidence of oral ulcers was reduced when dietary modifications (sprouts of chickpeas, wheat, green gram [mung bean], fenugreek seeds) and multivitamin supplementation were introduced in the diet on a regular basis [21].

It appears that oral tissue and the presence of cold and humidity could be the reasons for the entry of respiratory pathogens and their arrival in the lower airway. Fourrier et al. confirmed that bacteria causing pneumonia were first found in dental plaques [24]. At the same time, Scannapieco et al. revealed that the oral microbiota becomes modified by the use of antibiotics. It favors oral colonization with respiratory pathogens [54]. The most abundant genera in the oral cavity reported are *Haemophilus, Streptococcus, Prevotella*, and *Veillonella* (Human Microbiome Project 2012). In our study, *Pseudomonas* was abundant, a ubiquitous microorganism known for its environmental versatility and predominant opportunistic character. A problem arises when AmpC-(an enzyme conferring resistance to certain antibiotics) producing *Pseudomonas* spp., are isolated from asymptomatic professionals who disseminate the pathogen through droplets of saliva. The asymptomatic *Pseudomonas* may form a biofilm leading to lower respiratory tract infection in the cold and humid weather of Antarctica thus reducing the immunity. This enhanced abundance of *Pseudomonas* spp. in the saliva of participants during the Antarctica expedition is being reported for the first time by us. The finding could be a matter of concern for expedition management. Therefore, it is very important to establish control strategies and enlighten people to prevent bacterial dissemination. However, reporting of the oropharyngeal colonization of participants in the Indian expeditions have been scant so far. It could be because better hygiene and air circulation management have been established.

It is recognized that *Streptococcus* spp. promote recruitment of dental plaque bacteria through the production of extracellular glucan and allow co-aggregation with other germs and start the process of colonization [55]. It is known that species of streptococci are also associated with development of autoimmune diseases like meningitis, rheumatic fever, rheumatic [56,57]. Thus, the enhanced abundance of streptococci could be a probable reason for off and on reporting of joint pains by Antarctica expedition participants.

Evidences show the presence of lactobacilli in 100% of sampled children [58,59]. One factor that could influence the rate of salivary lactobacilli during childhood is the carbohydrate intake [60,61]. It is worthy to note that Antarctica expeditions are provided with an excessive supply of carbohydrates as dry fruits, chocolates, rice, etc., and that could be the possible reason for the increase in abundance of lactobacilli throughout the expedition. A correlation has been implicated in dryness of mouth and the salivary *Lactobacillus* count [62], which has been further associated with the symptoms of nervous breakdown. Therefore, it could be inferred that one of the probable factors for depression or mood swings in Antarctica participants could be diet as depressed subjects tend to eat a lot of sweet products, leading to enhanced abundance of lactobacilli. Conversely, lactobacilli also play an important role in inhibiting dental carries, which could be the possible reason for less reporting of dental carries by the participants [63,64]. The role of *Lactobacillus acidophilus* and *casei* as probiotics in improving the oral health by removing other harmful lactobacilli in dental plaque and saliva has also been reported as a preventive measure [65]. The dominant source of the salivary microbiome is most likely bacterial communities on the mucosal surfaces, especially the tongue dorsum, considering the similarities among microbiota compositions at various oral sites and in saliva. Therefore, it is reasonable to expect that the relative abundances of predominant bacteria in saliva are not directly associated with the quantity of dental plaque or dental health [66–68].

In humans, 24 h daylight does not cause much problem but long polar nights cause forgetfulness, affect thyroid function and sleep pattern. Disturbance in the circadian clock is a very important indicator of host inability to prevent opportunistic infections [69]. Edlund et al. have reported production of an N-actylserotonin-like molecule, an intermediate in the melatonin production, from the oral bacterial community [70]. This finding can be very well correlated with the altered oral microbiome of the Antarctica expedition participants with respect to their circadian rhythm and psychology in addition to long light and dark cycles.

Alteration in the metagenome affects the metabolism of both the commensal microbiota as well as the host microbiota, which eventually influences the human health leading to various diseases. The genes over the metagenome encode a large range of metabolites, proteins and carbohydrates, which do not solely affect the commensal microbiota but also the metabolism and immunity of the host. Thus, any alteration in the microflora is a factor of concern leading to disturbance in the metabolic pathways.

The relationship between the microbiota, voyage and stay in Antarctica has not been explored yet. The present study clearly elucidates the effects of the voyage and Antarctica conditions on the structure and metabolic conditions of the oral commensal microbes by sequencing 16S rRNA and WGS. All the pathways with altered expression in 1, 2 and 3-tiers are significant and positively correlated to metagenome and picrust with ‘r’score more than 0. In tier-1 positively correlated pathways to the metagenome are related to metabolism (cofactors and vitamins, amino acids, lipids, carbohydrates, glycol and its biosynthesis, and xenobiotics and its biodegradation), and organismal system (digestive system). In tier-2 specifically, in carbohydrate metabolism Galactose, Glycolysis/Gluconeogenesis and pyruvate were categorically affected, whereas in lipid metabolism, biosynthesis of lipids and fatty acids, their metabolism and elongation in mitochondria were predominantly affected. Similarly, other affected pathways like lipopolysaccharide, protein and peptidoglycan biosynthesis; in metabolism of amino acids, D-alanine metabolism have the potential to induce inflammation. Genes involved in nutrient metabolism and detoxification were also affected which could be due to insufficient nutrient intake because of severe seasickness including nausea, headache, stomach upset resulting in loss of appetite, restlessness, etc..

It is possible that cycling bacterial communities of the human microbiota produce the ideal number of active molecules, hormones, precursors, enzymes or metabolites for regulation of host metabolism, energy expenditure or host circadian rhythmicity. But many times, it happens that the activity changes may not be in full agreement with phylogeny. Some species differentially express pathways clustered with other genera. It needs an in-depth analysis to fully understand the phylogenetic relatedness between microbe and metabolic analysis. By using a bio-informative analytic approach based on the annotation of individual oral bacterial isolates, we identified unique alteration of the microbiota over time, which suggests that the oral microbiome has a dynamic potential involving regulation of community succession and cell-to-host-to-bacterium signaling interactions. Incidentally, very little information exists on the role of the oral microbiome and the underlying drivers of the differential pathways linked to taxonomic units. For hundreds of years, scorbutus has been reported to be caused by the commensal microbiome. Thus, the fact that only a few could be annotated highlights that this area of microbiome research remains a black box and needs significant attention to provide a deeper understanding of key interactions between the host and its microbiome.

The findings above indicate that exposure to the voyage and Antarctica environment can reasonably be assumed to be the factors causing reduced microbial diversity in the participants. Therefore, necessary steps for improved management strategies for better health conditions during expedition involve a well-planned voyage considering the entire expedition and sailing preparations with respect to nutrition and period and route of the expedition. Recommendations for the entire expedition include adding a protein-rich diet, intervention of probiotics to the food inventory, docking as often as possible to allow sailors to have contact with an environment with higher biodiversity, and thus enable the individual microbial diversity to be maintained at a relatively high level [1]. It is also noteworthy that a large number of organisms remained unclassified due to entirely different microbiota of the Antarctica continent compared to that of the earth’s surface. Therefore, it is emphasized that an in-depth analysis of the gut and oral microbiota of participants at the unique Antarctica is the need of the day.

## Conclusions

The microbial communities are bound to impact the health of the human host, and a better understanding of their dynamic complexity may contribute to the next level in medical diagnostic tools for the understanding of diseases. In the present study, we employed both 16S rRNA and WGS to understand the impact of a long voyage and stay in Antarctica, on the oral commensal microbiota of participants who underwent highly intense work, altered diet and circadian biorhythms in an extremely cold, isolated, humid and salty environment. Our data illustrated that the microbial diversity was reduced, several pathogens appeared and disappeared, altering carbohydrate, lipid and amino acid metabolisms. There was a higher abundance of the genera *Pseudomonas* and *Lactobacillus*, indicative of respiratory and oral diseases. Taxa analysis showed a large number of unclassified bacteria too. The influence of a long voyage to Antarctica on human health by examining the commensal microbiota using metagenomic approaches has been reported here for the first time. Future work will focus on the exact factors in the voyage and stay in Antarctica, that cause changes in the commensal microbiota on the basis of metagenomic analysis carried out more extensively on a larger sample size.
