# Supplementary material for: Diversity and functional analysis of salivary microflora of Indian Antarctic expeditionaries
Source: J Oral Microbiol. 2019 Feb 27;11(1):1581513. doi: 10.1080/20002297.2019.1581513 (PMC6394331; doi:10.1080/20002297.2019.1581513)
Supplement: Supplemental Material [file ZJOM_A_1581513_SM6877.doc]

**SUPPLEMENTARY DATA**

**16S Data Statistics**

**Table : (ST1)**

| **Name** | **Number of reads** | **Avg.length** | **Number of reads after trim** | **Percentage trimmed** | **Avg.length after trim** |
| --- | --- | --- | --- | --- | --- |
| A1 | 164374 | 523.8 | 164374 | 100% | 523.7 |
| A10 | 127853 | 410 | 127853 | 100% | 396.8 |
| A11 | 124815 | 411.8 | 124815 | 100% | 390 |
| A12 | 133867 | 409.8 | 133867 | 100% | 395.6 |
| A2 | 145568 | 507.6 | 145568 | 100% | 507.5 |
| A3 | 154971 | 516.2 | 154971 | 100% | 516.1 |
| A4 | 110766 | 509.4 | 110766 | 100% | 509.3 |
| A5 | 120169 | 520.3 | 120169 | 100% | 520.3 |
| A6 | 75964 | 512.9 | 75964 | 100% | 512.8 |
| A7 | 109749 | 411.3 | 109749 | 100% | 392.8 |
| A8 | 101415 | 410.6 | 101415 | 100% | 392 |
| A9 | 103238 | 409.6 | 103238 | 100% | 389 |
| B1 | 133006 | 505.3 | 133006 | 100% | 505.3 |
| B10 | 132588 | 410.5 | 132588 | 100% | 396.6 |
| B11 | 122062 | 409.7 | 122062 | 100% | 397.5 |
| B12 | 138850 | 411 | 138850 | 100% | 396.2 |
| B2 | 94584 | 516.1 | 94584 | 100% | 516 |
| B3 | 122118 | 508 | 122118 | 100% | 508 |
| B4 | 127562 | 506.6 | 127562 | 100% | 506.6 |
| B5 | 108351 | 511.5 | 108351 | 100% | 511.5 |
| B6 | 94342 | 517.4 | 94342 | 100% | 517.4 |
| B7 | 132473 | 408.7 | 132473 | 100% | 395.8 |
| B8 | 121543 | 409.2 | 121543 | 100% | 396.4 |
| B9 | 133788 | 409.4 | 133788 | 100% | 396.2 |
| C1 | 138404 | 529.5 | 138404 | 100% | 529.4 |
| C10 | 119819 | 408.6 | 119819 | 100% | 394 |
| C11 | 123332 | 409.4 | 123332 | 100% | 396.3 |
| C12 | 117482 | 409.7 | 117482 | 100% | 397.1 |
| C2 | 135258 | 514.8 | 135258 | 100% | 514.7 |
| C3 | 123112 | 527.3 | 123112 | 100% | 527.2 |
| C4 | 170860 | 508.9 | 170860 | 100% | 508.8 |
| C5 | 147682 | 513.3 | 147682 | 100% | 513.2 |
| C6 | 159271 | 510.9 | 159271 | 100% | 510.8 |
| C7 | 115556 | 410.8 | 115556 | 100% | 391.7 |
| C8 | 134000 | 409.4 | 134000 | 100% | 392 |
| C9 | 137742 | 410.2 | 137742 | 100% | 397 |

**Figure S2**


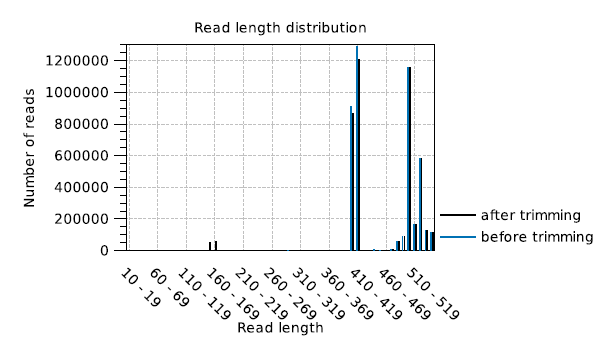


**Figure S2: sequence read length before/after trimming**

**Table : ST2**

**OTU Statistics:**

| **Sample** | **Total number of reads** | **Filtered or chimeric reads** | **Reads in OTUs** |
| --- | --- | --- | --- |
| A1 | 163994 | 12358 | 151636 |
| A10 | 119171 | 56183 | 62988 |
| A11 | 113301 | 37865 | 75436 |
| A12 | 125228 | 65624 | 59604 |
| A2 | 145354 | 10990 | 134364 |
| A3 | 154674 | 11674 | 143000 |
| A4 | 110592 | 9345 | 101247 |
| A5 | 119912 | 10029 | 109883 |
| A6 | 75830 | 7700 | 68130 |
| A7 | 100791 | 43576 | 57215 |
| A8 | 92406 | 37071 | 55335 |
| A9 | 92755 | 30812 | 61943 |
| B1 | 132774 | 10696 | 122078 |
| B10 | 120581 | 64265 | 56316 |
| B11 | 113866 | 45956 | 67910 |
| B12 | 130065 | 54953 | 75112 |
| B2 | 94258 | 10162 | 84096 |
| B3 | 121798 | 13030 | 108768 |
| B4 | 127286 | 11863 | 115423 |
| B5 | 108028 | 11596 | 96432 |
| B6 | 94178 | 8170 | 86008 |
| B7 | 122798 | 66306 | 56492 |
| B8 | 111029 | 61936 | 49093 |
| B9 | 121291 | 68547 | 52744 |
| C1 | 138068 | 9814 | 128254 |
| C10 | 107275 | 47670 | 59605 |
| C11 | 114605 | 63013 | 51592 |
| C12 | 110190 | 50984 | 59206 |
| C2 | 134946 | 13086 | 121860 |
| C3 | 122764 | 11564 | 111200 |
| C4 | 170490 | 11928 | 158562 |
| C5 | 147348 | 11582 | 135766 |
| C6 | 158919 | 11755 | 147164 |
| C7 | 106219 | 49325 | 56894 |
| C8 | 121578 | 68512 | 53066 |
| C9 | 129727 | 42859 | 86868 |

**Figure S3**


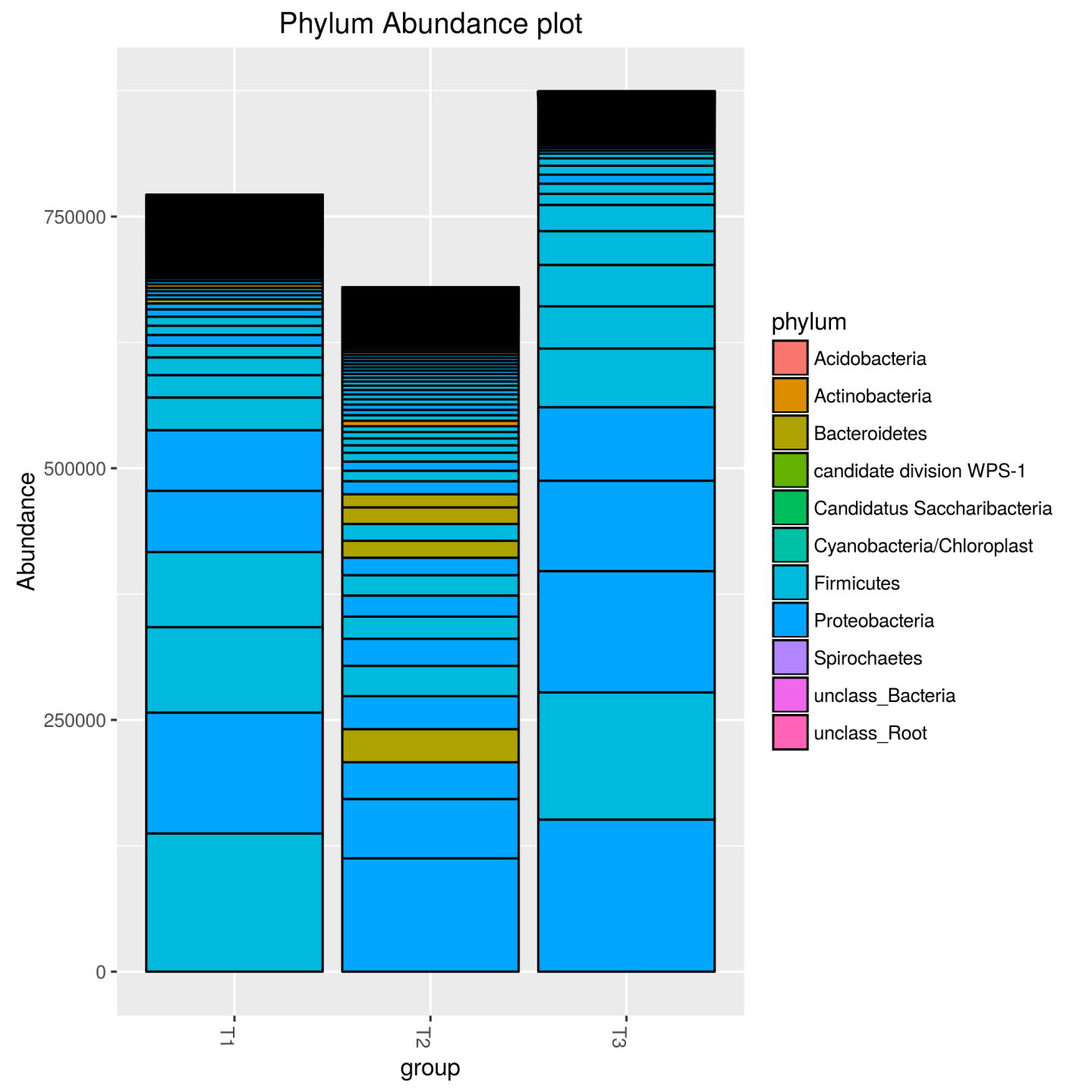


**Figure S3: Phylum abundance plot stacked for top 11 phyla’s (Values (number of sequences) are stacked in order in each bar, with the greatest at the bottom)**

**Figure S4**


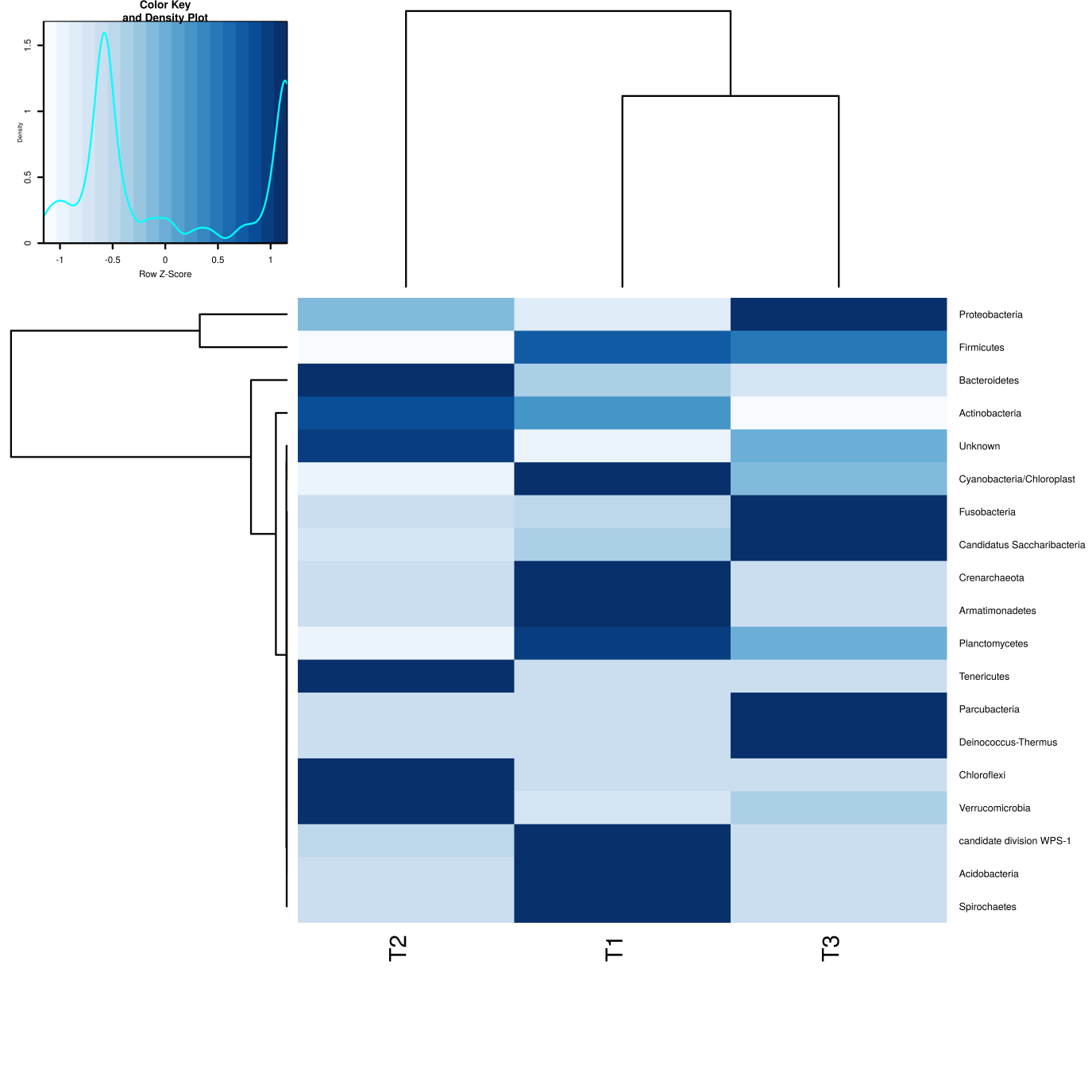


**Figure S4: Heatmap Phylum group for each group (a two dimensional data representation in which values are represented as colors)**

**Figure S5**


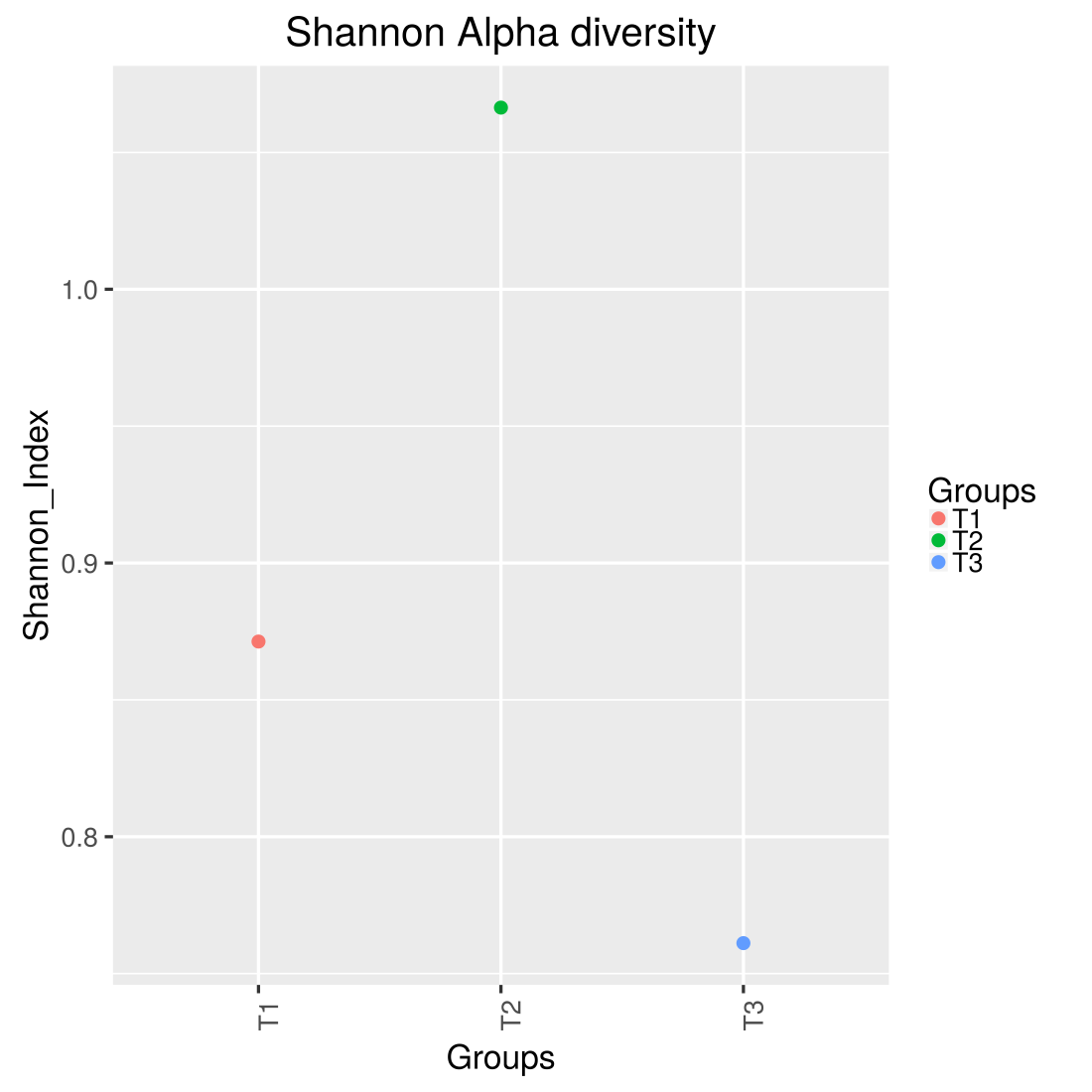


**Figure S5: Alpha Diversity Shannon plot (Represents richness estimate of each sample)**

**SALIVA CLASS LEVEL**

**Figure S6**


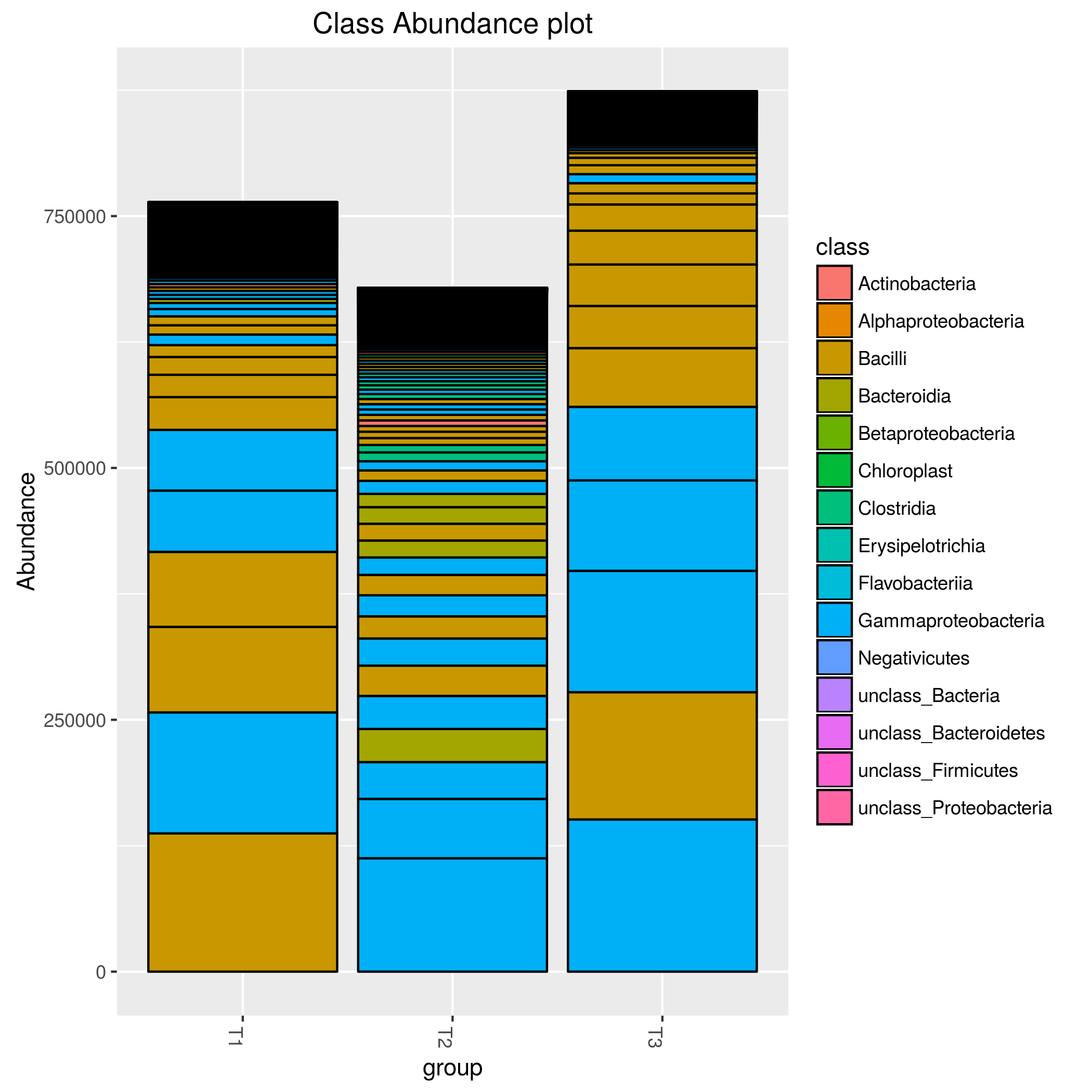


**Figure S6: Class abundance plot stacked for top 15 class (Values (number of sequences) are stacked in order in each bar, with the greatest at the bottom)**

**Figure S7**


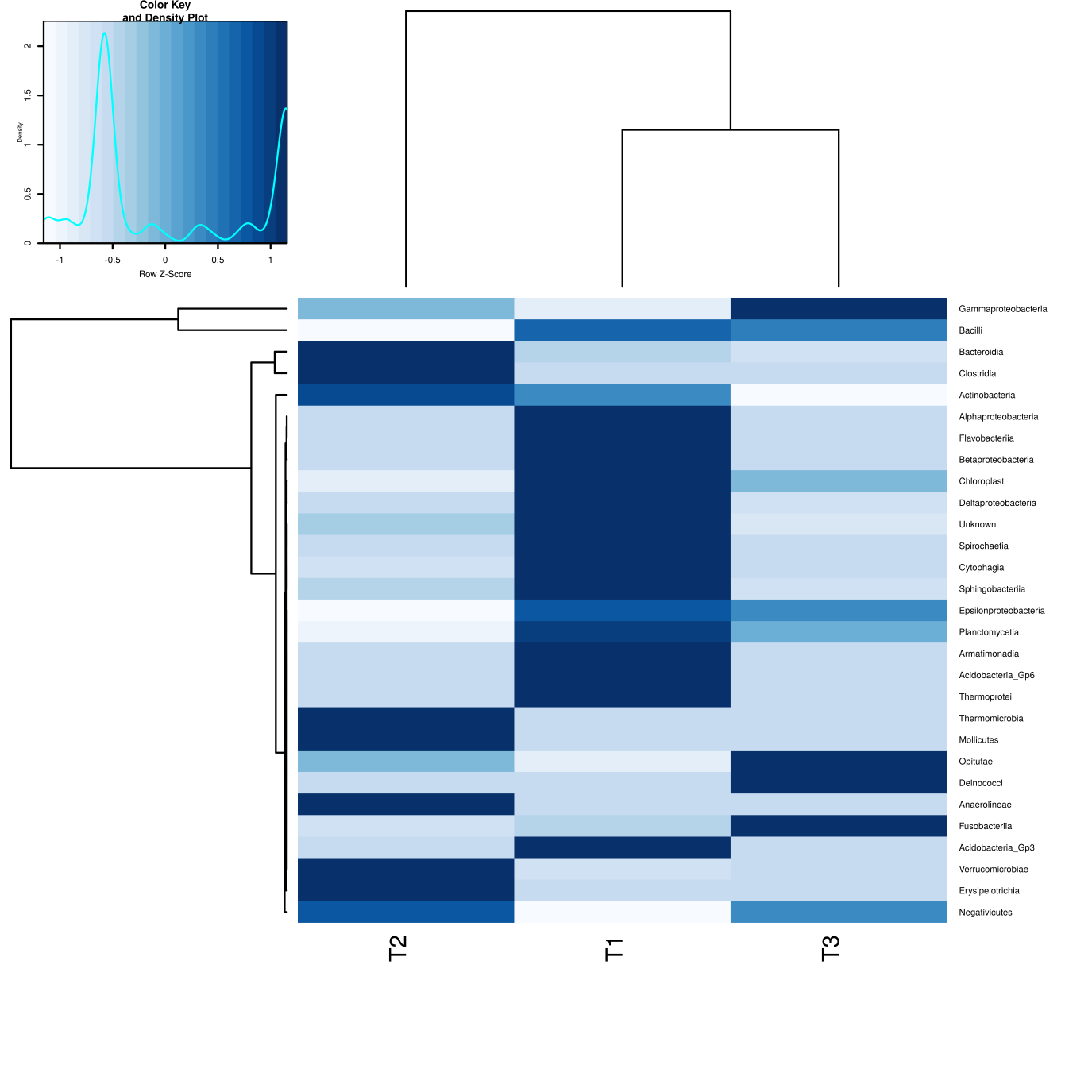


**Figure S7: Heatmap class for each group (a two dimensional data representation in which values are represented as colors)**

**Figure S8**


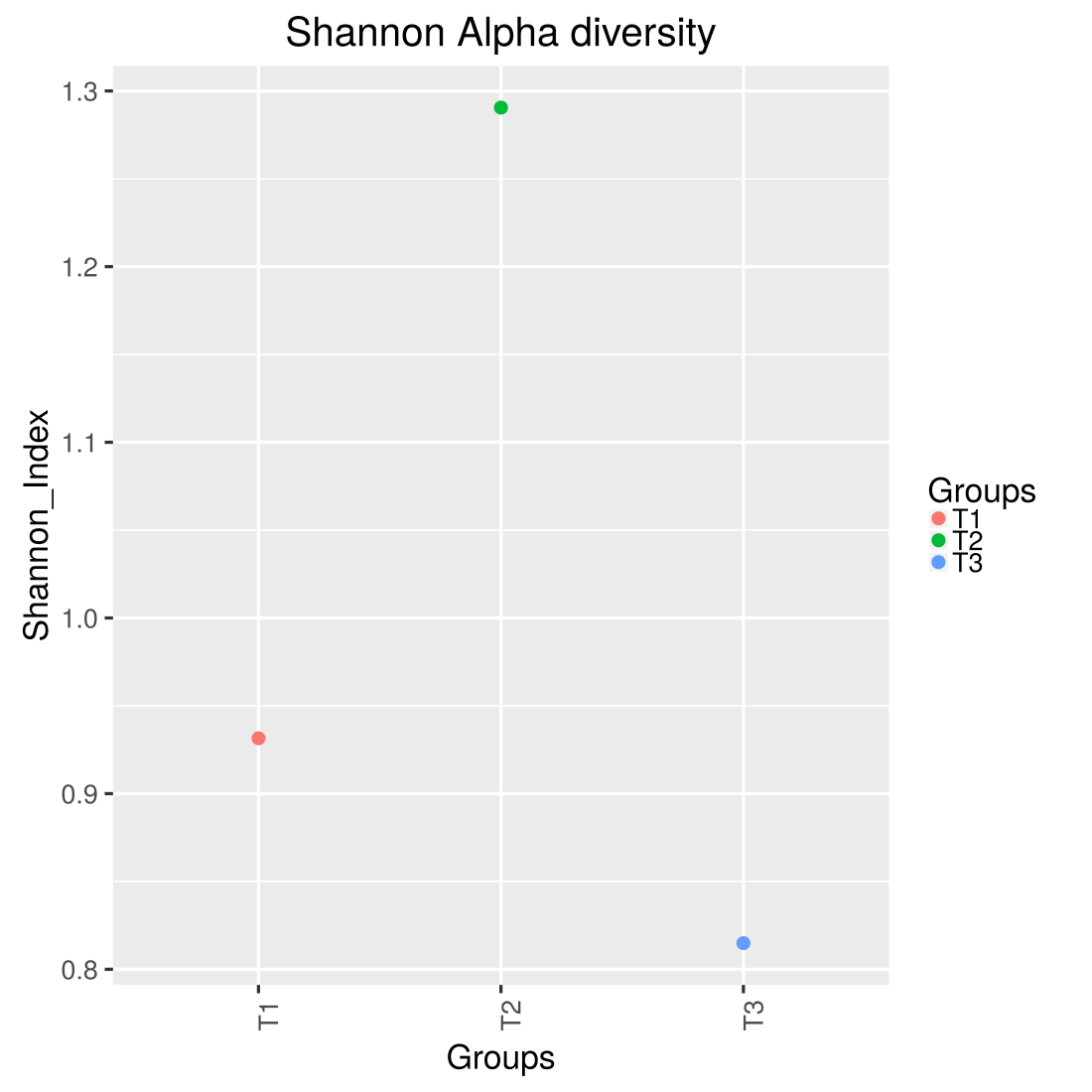


**Figure S8: Alpha Diversity Shannon plot (Represents richness estimate of each sample)**

**Figure S9**

**Alignment tree circular Cladogram**


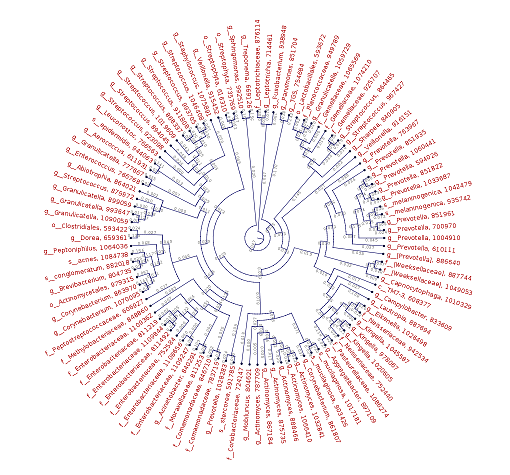


**Figure S10**

**PCoA :**


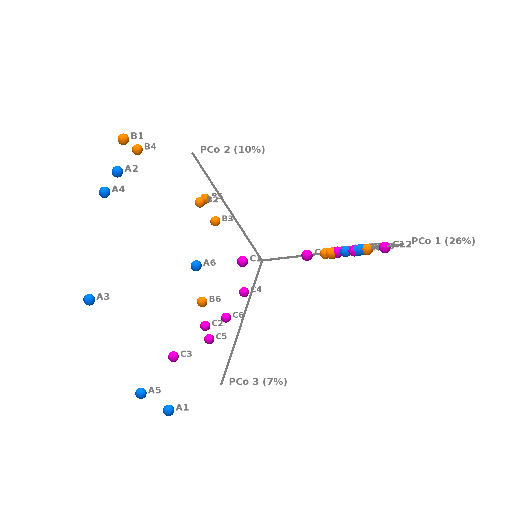


**Bray-Curtis**


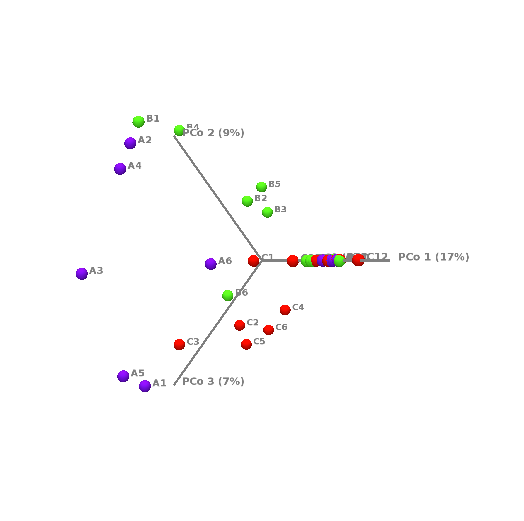


**Jaccard**

**Table ST4**

**Whole genome shotgun Metagenomics:**

**Raw data Statistics**

| **Sample Name** | **Number of Sequences** | **Sequence length** | **GC%** |
| --- | --- | --- | --- |
| T1_Saliva_1 | 14377747 | 75 | 45 |
| T1_Saliva_2 | 14377747 | 75 | 45 |
| T2_Saliva_1 | 16567256 | 75 | 43 |
| T2_Saliva_2 | 16567256 | 75 | 43 |
| T3_Saliva_1 | 17780631 | 75 | 43 |
| T3_Saliva_2 | 17780631 | 75 | 43 |

**Table ST5**

De novo Assembly Statistics:

| **Kmer** | **N50** | **No. of seq** | **Total Bases** |
| --- | --- | --- | --- |
| 21 | 525 | 304525 | 155107584 |
| 61 | 504 | 196048 | 96275571 |

**Based on N50 and number of sequences denovo assembly at Kmer = 21 was used for further analysis**

**Table ST6**

**Annotation Statistics:**

CDS Annotation with BLAST:

| **Kmer** | **Number of CDSs annotated with a BLAST hit** | **Percent of CDS annotated with a BLAST hit** |
| --- | --- | --- |
| 21 | 87843 | 28.31% |

**Table ST7**

**CDS Annotation with Pfam domain and GO terms:**

| **Kmer** | **Total number of Pfam domains identified** | **Number of CDS containing a Pfam domain** | **Percent of CDS containing a Pfam domain** | **Total number of GO terms added** | **Number of CDS containing a Pfam domain annotated with a GO term** | **Percent of CDS containing a Pfam domain annotated with a GO term** |
| --- | --- | --- | --- | --- | --- | --- |
| 21 | 101976 | 89916 | 28.98% | 101029 | 56264 | 18.13% |

**Table ST8**

Functional Profile:

| **Sample** | **Number of Pfam domains with at least one match** | **Number of GO terms with at least one match** | **Number of BLAST hits with at least one match** | **Number of matches to Pfam domains** | **Total number of matches to GO terms** | **Total number of matches to a BLAST hit** |
| --- | --- | --- | --- | --- | --- | --- |
| T1 | 5189 | 2400 | 34657 | 839979 | 8012729 | 1834166 |
| T2 | 5189 | 2400 | 34657 | 2566222 | 28017134 | 3426069 |
| T3 | 5189 | 2400 | 34657 | 5154526 | 57384257 | 5698024 |

**Figure S11**

**Pfam Abundance Plot:**


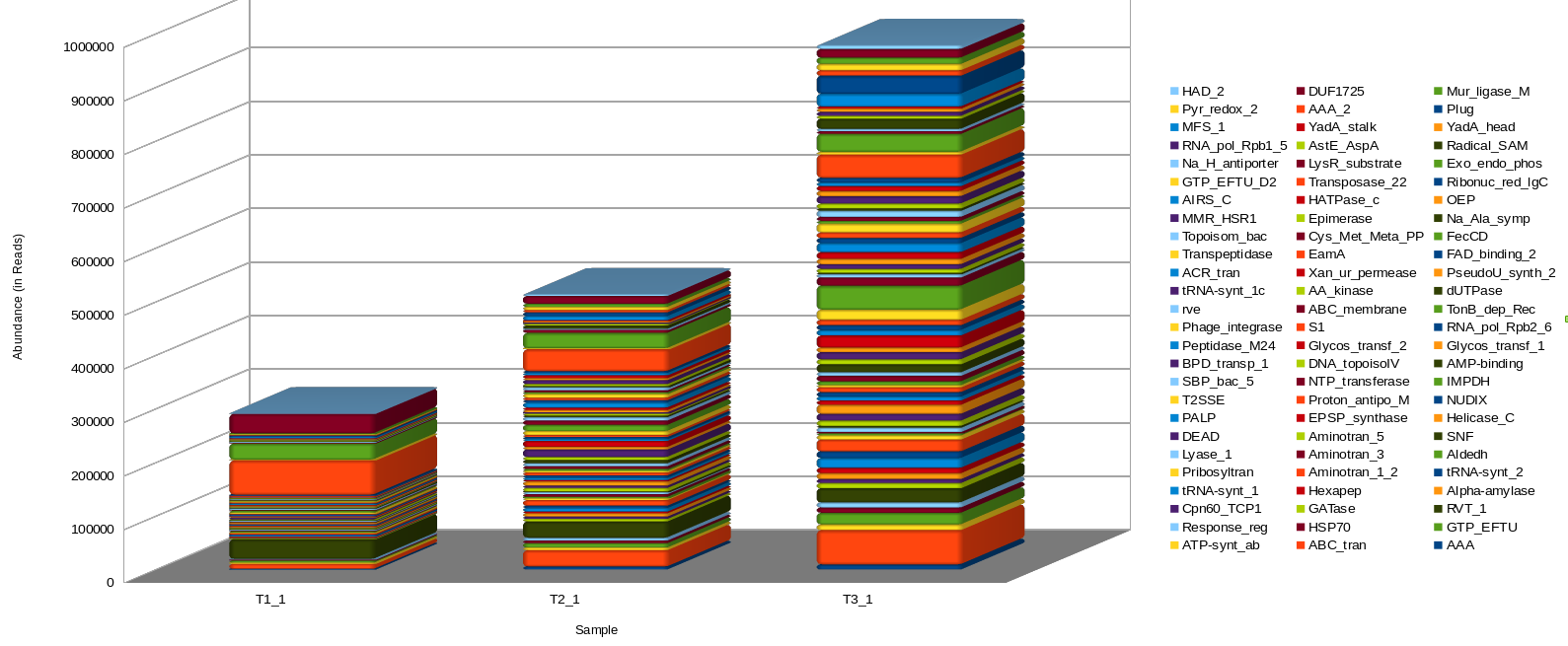


**Abundance corresponding to different Pfam Domain**

**
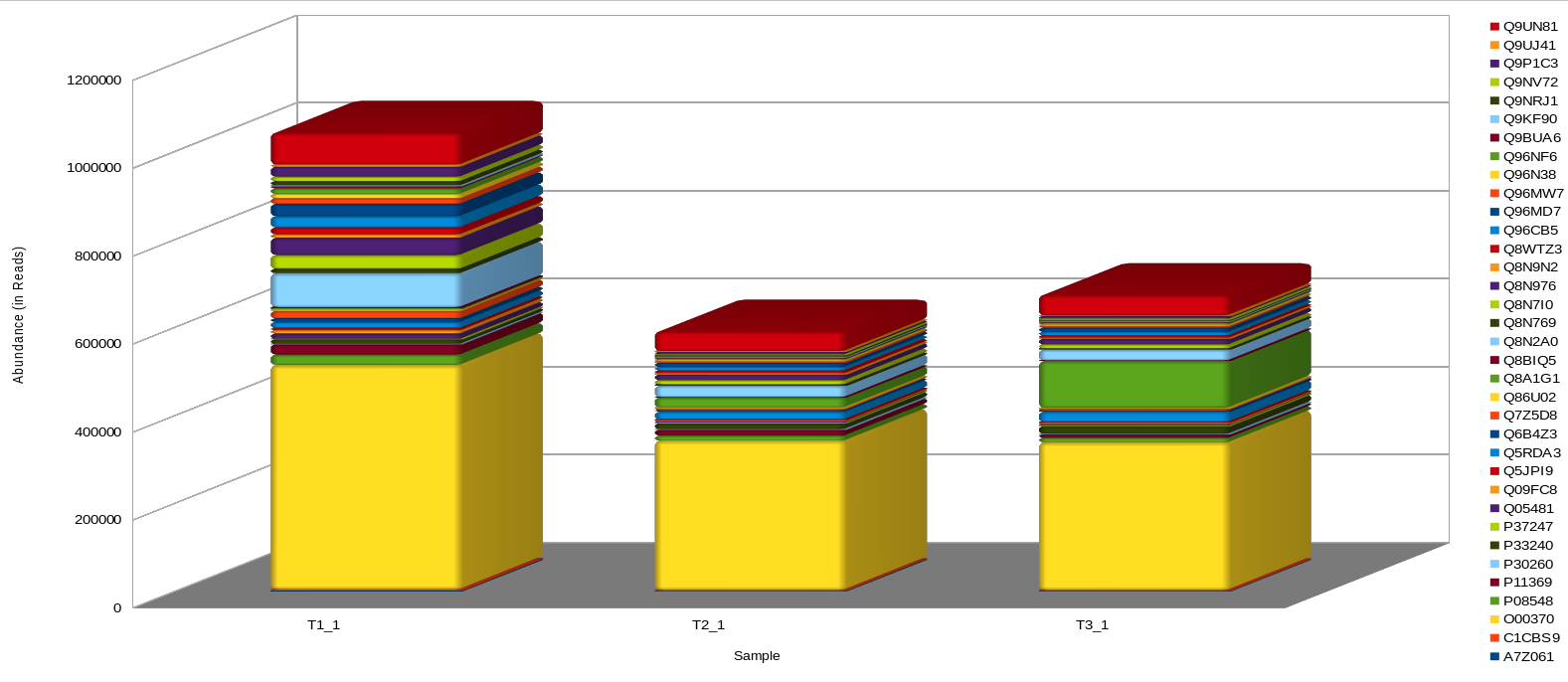
**

**Abundance corresponding to best Blast hits**

**Figure S12**

**Gene Ontology Based on Combined Abundance of Samples**


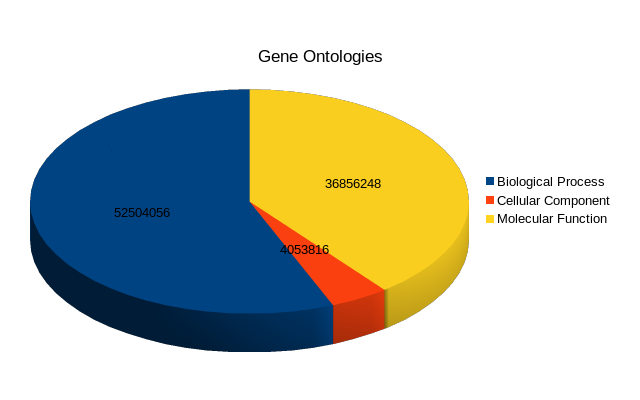


**Gene Ontology Based on number of different GO terms**
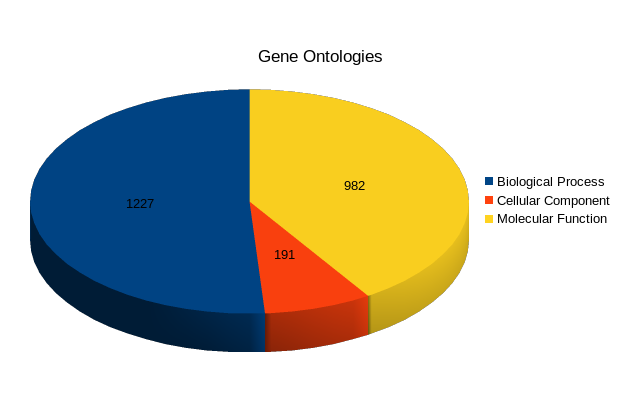


**Table ST9**

Pathway Statistics:

| **Combination** | **Total pathway annotated genes** | **Differential expressed pathway annotated genes (log2FC)** | **Significant pathway annotated genes (pval <=0.1)** |
| --- | --- | --- | --- |
| **T1_T2** | **18505** | **10921** | **148** |
| **T1_T3** | **18446** | **11364** | **35** |
| **T2_T3** | **18792** | **5896** | **1213** |

**Figure S13**


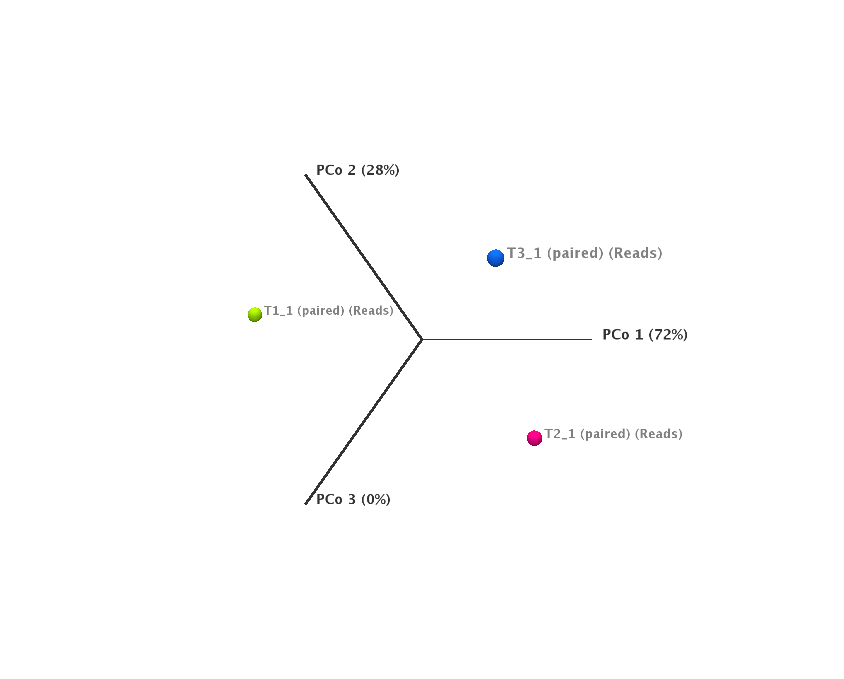
**WGS PcoA Plots:**

**
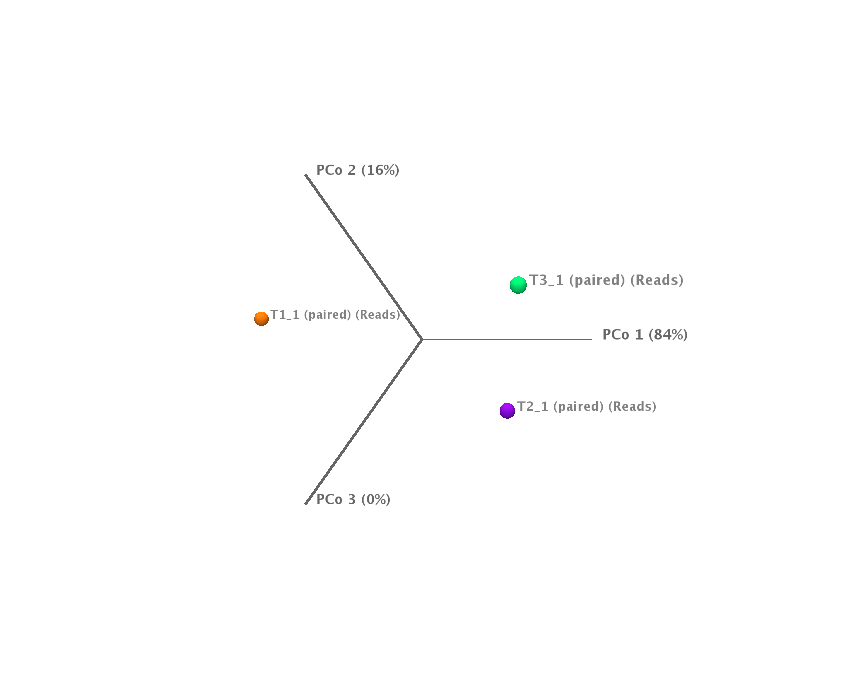
**

**Jaccard Bray-Curtis**
